# TOR Complex 2- independent mutations in the regulatory PIF pocket of Gad8^AKT1/SGK1^ define separate branches of the stress response mechanisms in fission yeast

**DOI:** 10.1371/journal.pgen.1009196

**Published:** 2020-11-02

**Authors:** Emese Pataki, Luba Simhaev, Hamutal Engel, Adiel Cohen, Martin Kupiec, Ronit Weisman

**Affiliations:** 1 Department of Natural and Life Sciences, The Open University of Israel, Ra'anana, Israel; 2 Blavatnik Center for Drug Discovery, Tel Aviv University, Tel Aviv, Israel; 3 The Shmunis School of Biomedicine & Cancer Research, Tel Aviv University, Ramat Aviv, Tel Aviv, Israel; University of Oslo, NORWAY

## Abstract

The Target of rapamycin (TOR) protein kinase forms part of TOR complex 1 (TORC1) and TOR complex 2 (TORC2), two multi-subunit protein complexes that regulate growth, proliferation, survival and developmental processes by phosphorylation and activation of AGC-family kinases. In the fission yeast, *Schizosaccharomyces pombe*, TORC2 and its target, the AGC kinase Gad8 (an orthologue of human AKT or SGK1) are required for viability under stress conditions and for developmental processes in response to starvation cues. In this study, we describe the isolation of *gad8* mutant alleles that bypass the requirement for TORC2 and reveal a separation of function of TORC2 and Gad8 under stress conditions. In particular, osmotic and nutritional stress responses appear to form a separate branch from genotoxic stress responses downstream of TORC2-Gad8. Interestingly, TORC2-independent mutations map into the regulatory PIF pocket of Gad8, a highly conserved motif in AGC kinases that regulates substrate binding in PDK1 (phosphoinositide dependent kinase-1) and kinase activity in several AGC kinases. Gad8 activation is thought to require a two-step mechanism, in which phosphorylation by TORC2 allows further phosphorylation and activation by Ksg1 (an orthologue of PDK1). We focus on the Gad8-K263C mutation and demonstrate that it renders the Gad8 kinase activity independent of TORC2 *in vitro* and independent of the phosphorylation sites of TORC2 *in vivo*. Molecular dynamics simulations of Gad8-K263C revealed abnormal high flexibility at T387, the phosphorylation site for Ksg1, suggesting a mechanism for the TORC2-independent Gad8 activity. Significantly, the K263 residue is highly conserved in the family of AGC-kinases, which may suggest a general way of keeping their activity in check when acting downstream of TOR complexes.

## Author summary

Protein kinases catalyze the transfer of phosphate from high-energy, phosphate-donating molecules, such as ATP, to their substrates. This process is pivotal for regulation of almost any aspect of cellular biology. Many human diseases are associated with aberrant functions of protein kinases due to mutations. Accordingly, there is a growing number of kinase inhibitors that have been approved for clinical use. A better understanding of how protein kinases become active and how their activity is relayed to regulate their cellular functions is much needed for rational design of kinase inhibitors and for their optimal use in the clinic. The AGC-family of protein kinases play key roles in regulating cellular growth, proliferation and survival. In human cells, as well as in the fission yeast, our cellular model system, a subgroup of the AGC kinases is activated by the TOR protein kinases. Here we report the isolation of mutations in the AGC kinase Gad8 (AKT or SGK1 in human) that bypass the requirement for activation by TOR. Analyses of how these mutations affect cellular growth revealed separate branches of stress response mechanisms downstream of Gad8, while computer simulation methods suggested a molecular mechanism that keeps the activity of Gad8 in check.

## Introduction

Target of rapamycin (TOR) is an atypical serine/threonine protein kinase that belongs to the family of phosphatidylinositol-3 kinase related kinases. TOR kinases are highly conserved throughout evolution and control several aspects of metabolism, cellular growth and survival. Consequently, deregulation of TOR activity is associated with pathological outcomes, including cancer, diabetes and neurological defects [1–3]. TOR kinases are found in two highly conserved complexes, known as TORC1 and TORC2, which were first identified in the budding yeast *Saccharomyces cerevisiae* [4, 5]. The mammalian TORC1, mTORC1, which contains the mTOR kinase together with the Raptor protein, is well-known for its positive role in promoting anabolic processes, while also inhibiting catabolic and starvation responses [3, 6]. These TORC1 cellular functions are conserved in model organisms, including the budding yeast, *Saccharomyces cerevisiae* and the fission yeast, *S*. *pombe* [5, 7]. The mammalian TORC2, mTORC2, is characterized by TOR together with the Rictor and mSin1 proteins and acts downstream of PI3K/insulin signaling to regulate cellular growth and metabolism [1, 8]. The cellular roles of the TORC2 complexes and the extent of their evolution conservation are less well-understood compared with those of TORC1 [7]. The subcellular localization of TORC2 is also a subject for ongoing research. Like in higher eukaryotes, *S*. *pombe* TORC2 has been localized to the plasma membrane, but it is also found in the nucleus (reviewed in [9]).

TOR complexes mediate many of their functions via phosphorylation and activation of downstream AGC kinases, a group that was named after three representative families, the cAMP- dependent protein kinase A (PKA), the cGMP-dependent protein kinase (PKG) and the protein kinase C (PKC) families [10]. mTORC1 directly phosphorylates and activates the p70 ribosomal S6 kinase-1 (S6K1), which subsequently regulates growth, metabolism and translation [11–13]. The *S*. *cerevisae* orthologue of S6K1 is Sch9 [14], while three AGC kinases, Psk1, Sck1 and Sck2, act downstream of the *S*. *pombe* TORC1 [15]. mTORC2 mediates its effects via phosphorylation and activation of three different members of the AGC kinase family: the three isoforms of AKT (also known as PKB) [16–18], SGK1 [19] and PKCα [20, 21]. In *S*. *cerevisiae*, there are three AGC kinases that act downstream of TORC2, Ypk1, Ypk2 and Pkc1, which are involved in a wide variety aspects of membrane homeostasis, regulation of the actin cytoskeleton, cell cycle progression and genome integrity ([22–30] and reviewed in [31, 32]). In *S*. *pombe*, TORC2 is responsible for the phosphorylation and activation of the AGC kinase Gad8 [33], thereby regulating several aspects of cell cycle progression, promoting survival under a large variety of stress conditions and enabling cells to enter the sexual development pathway in response to starvation [33–38]. Disruption of *gad8*^+^ results in a phenotype very similar to disruption of TORC2, including delayed entrance into mitosis, defects in growth under conditions of high temperature, osmotic, oxidative stress [33] or genotoxic stresses [39]. The activity of *S*. *pombe* TORC2 is acutely inhibited by glucose depletion or exposure to osmotic stress [40], but this inhibition is transient [41, 42]. TORC2-Gad8 activity is also regulated under nitrogen starvation and in response to quiescence [43, 44].

The majority of members of the AGC kinase that lie downstream of TOR complexes share a common mechanism of activation that involves phosphorylation at three major conserved sites (reviewed in [10, 45, 46]). One phosphorylation site is located at the activation loop (also known as the A-loop), which is also present in the kinase domain of other conventional protein kinases. Two additional sites are characteristic for AGC kinases and are located C- terminally to the kinase domain at the so called turn motif (TM) and hydrophobic motif (HM). Phosphorylation events at the C- terminal extension are mediated by TOR complexes, while the activation loop site is phosphorylated by PDK1 in higher eukaryotes, or by PDK1-like kinases: Pkh1 and Pkh2 in *S*. *cerevisiae* [47] or Ksg1 in *S*. *pombe* [33, 48]. Phosphorylation at the HM in several AGC kinases, including SGK1 and S6K1, but not AKT, leads to docking into a regulatory hydrophobic pocket in PDK1, called the PIF (PDK-interacting fragment) motif. This intermolecular interaction between PDK1 and its downstream target kinases facilitates substrate recruitment and induces the kinase activity of PDK1 towards the activation loop of downstream kinases [49–51]. Several AGC kinases, including S6K1, AKT and SGK1 employ the PIF pocket for intramolecular interaction with the phosphorylated hydrophobic motif, thus promoting their kinase activation [52, 53]. The role of the PIF pocket in allosteric regulation of the ATP-binding and kinase activity of PDK1 could provide valuable sites for selective inhibitors [54, 55].

Similar to other AGC-kinases, full activation of Gad8 requires phosphorylation at all three sites. The catalytic subunit of *S*. *pombe* TORC2, Tor1, is responsible for phosphorylation of S546 in the HM and S527 in the TM [33]. Previous studies suggested that TORC2-dependent phosphorylation at S546 facilitates the access of Ksg1 (*S*. *pombe* PDK1) to phosphorylate Thr387 in the activation loop [33]. In this study we demonstrate that under stress conditions, point mutations in the PIF pocket of Gad8 render its kinase activity independent of TORC2, possibly by modifying the protein conformation of the activation loop. Our studies suggest structural roles for specific amino acids in keeping Gad8 activity in check, while also identifying a separation of function of Gad8 activity under distinct sets of stress conditions.

## Results

### Isolation of Tor1-independent mutant alleles of *gad8*^+^

**Fig 1:**
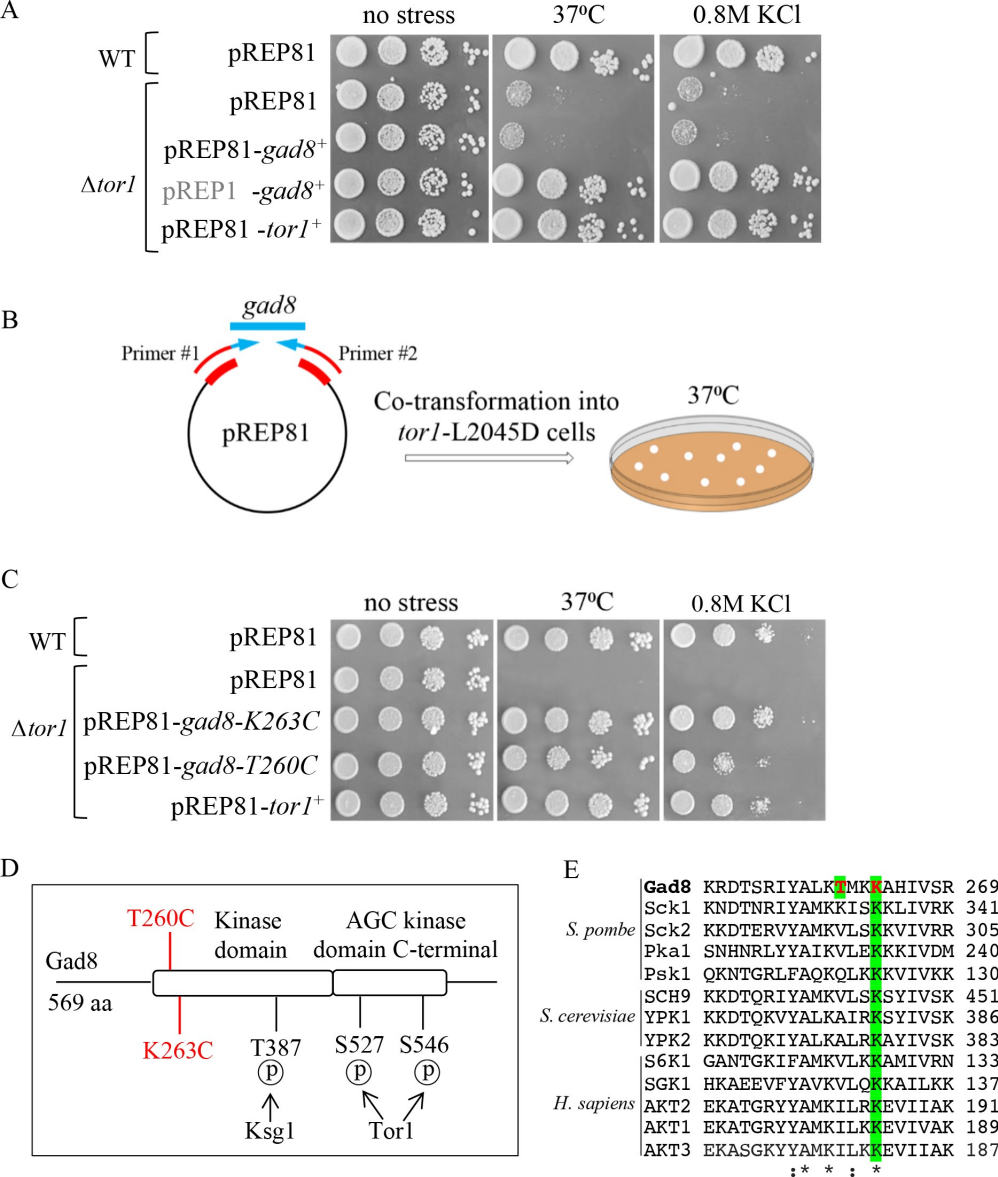
Isolation of *tor1*-independent alleles of *gad8*. ***A*,** Serial dilutions of wild-type (WT) and Δ*tor1* cells transformed with the indicated plasmids. pREP1, in grey, allows strong overexpression, while pREP81, in black, allows weak overexpression of the cloned gene. Cells were spotted onto minimal medium with, or without 0.8M KCl and were incubated for 4 days at 30°C (no stress) or at high temperature (37°C). ***B*,** Schematic representation of the screen carried out to isolate *tor1*-independent *gad8* alleles. The *gad8*^*+*^ open reading frame was amplified under PCR mutagenesis conditions using primers that contained sequences homologous to pREP81 sequences (in red) and sequences homologous to *gad8*^+^ (in blue). The mutagenized *gad8* fragments were co-transformed together with linearized pREP81 plasmids into the *tor1*-*L2045D* strain and transformants were selected on minimal plates at 37°C. ***C*,** Serial dilutions of wild-type (WT) and Δ*tor1* cells as described in A. ***D*,** Schematic representation of Gad8. Indicated in red are the *tor1*-independent *gad8* mutations, T260C and K263C, and in black the Ksg1-dependent phosphorylation site, T387, and the Tor1-dependent phosphorylation sites, S527 and S546. ***E*,** Alignment of the amino acid sequences surrounding the *T260C* and *K263C* point mutations in several members of the AGC kinases. The *tor1*-independent *gad8* mutations are in red. The K263, but not T260, residue is conserved in all the examined AGC kinases.

Tor1, the catalytic subunit of *S*. *pombe* TORC2, is responsible for the phosphorylation (and therefore activation) of the Gad8 kinase [33]. Consistent with previous studies that showed that strong overexpression of *gad8*^+^ suppressed phenotypes associated with Δ*tor1* [56], strong overexpression of *gad8*^+^ under the regulation of the *nmt1*^+^ promoter from the multicopy plasmid pREP1 [57] rescued the high temperature (37°C) or osmotic stress (KCl) sensitivities of Δ*tor1* cells (pREP1-Gad8, Fig 1A). Thus, Gad8 that lacks Tor1-dependent phosphorylation retains a certain level of activity when strongly overexpressed. In contrast, low overexpression of *gad8*^+^ from the pREP81 plasmid, which contains a weak version of the *nmt1*^+^ promoter [57] is unable to suppress the sensitivity of Δ*tor1* cells at high temperature or under osmotic stress conditions (pREP81-Gad8, Fig 1A). Therefore, we reasoned that the pREP81-*gad8*^+^ construct, in combination with random mutagenesis, could be useful for the isolation of TORC2 (Tor1)- independent *gad8* mutant alleles.

We obtained a library of mutant alleles of *gad8* by random mutagenesis using error-prone PCR with pREP81-*gad8*^+^ as template. We then co-transformated the resulting PCR fragments together with linearized pREP81 plasmids into yeast cells (see schematic presentation in Fig 1B). Since Δ*tor1* cells are poorly transformed, we used mutant cells harboring a point mutation in *tor1*^+^, *tor1-L2045D*, which renders the mutant cells sensitive to high temperature or to osmotic stress [58]. We screened 23,000 colonies and isolated 3 plasmids that suppressed the temperature-sensitive phenotype of *tor1-L2045D* at 37°C. We re-transformed these three plasmids, *pREP81-gad8-m1/m2/*m3, into naive *tor1-L2045D* or Δ*tor1* cells and found that they fully suppressed their high temperature and KCl sensitive phenotypes (S1A Fig). DNA sequencing indicated 5–9 mutations within the open reading frame (ORF) of *gad8*^*+*^ in each of the three isolated plasmids (S1B Fig). By a combination of sub-cloning and functional analysis, we identified two mutations, lysine 263 to cysteine (K263C) and threonine 260 to cysteine (T260C), which alone suppressed the temperature or KCl sensitive phenotypes of Δ*tor1* cells (Fig 1C). The K263C mutation was found in two independently isolated plasmids (*pREP81-gad8-m1* and *pREP81-gad8-m2*), while the T260C mutation occurred only once (*pREP81-gad8-m3*) (S1B Fig). The K263C and T260C mutations are located at the N-terminal edge of the kinase domain of Gad8 (Fig 1D). The K263 residue is highly conserved among members of the family of AGC kinases, while T260 is not (Fig 1E).

### The *gad8-K263C* or *gad8-T260C* mutations suppress a range of defects associated with the loss of TORC2

**Fig 2:**
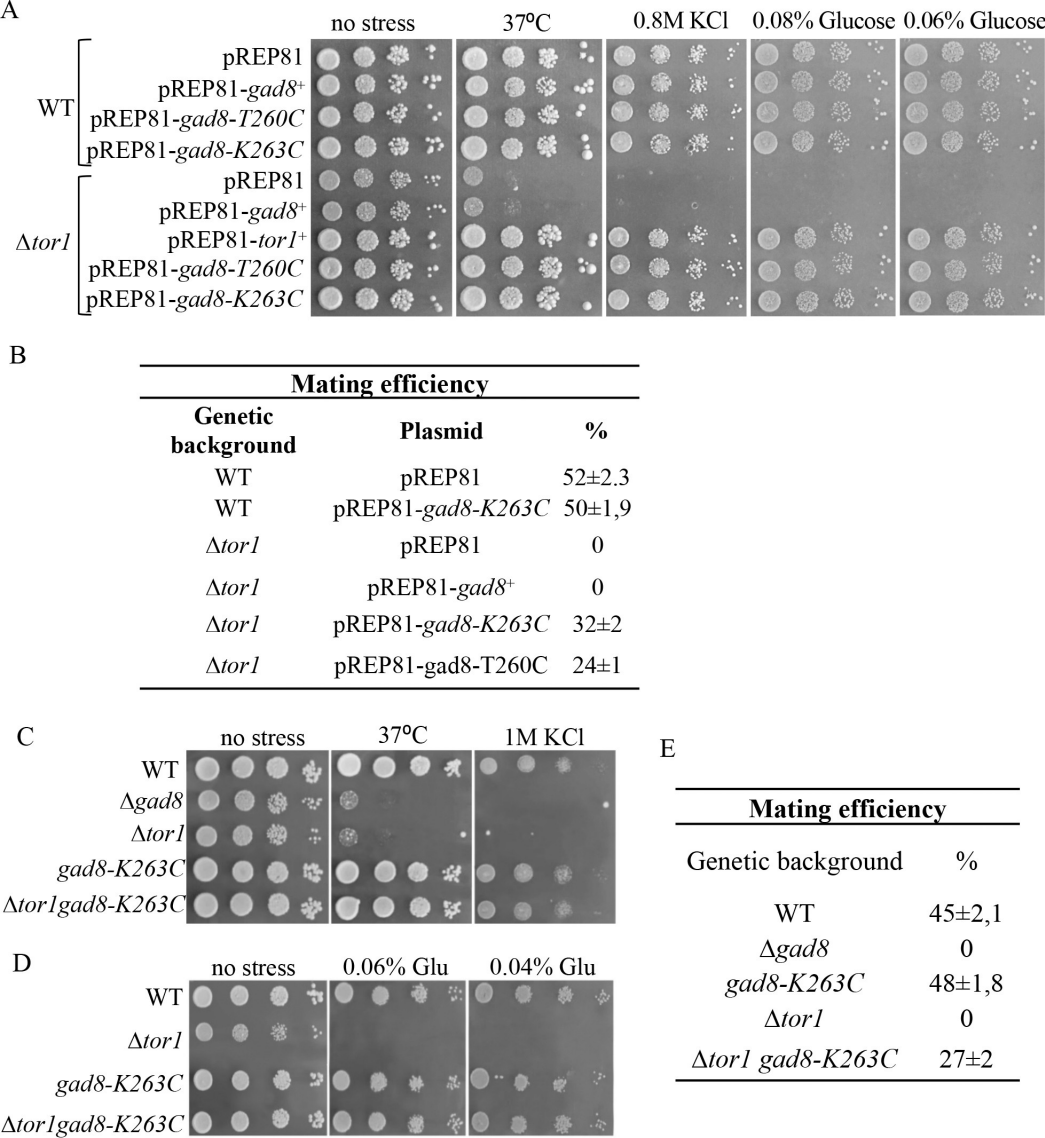
*gad8-K263C* or *gad8-T260C* suppress high temperature, osmotic stress, low-glucose and sterility in TORC2 mutant cells. ***A-B*,** Suppression by *gad8-K263C* and *gad8-T260C* when expressed from multicopy plasmids. Serial dilutions of wild-type (WT), and Δ*tor1* cells transformed with the indicated plasmids. Cells were spotted onto minimal medium at 30°C (no stress), 37°C, or at 30°C with 1M KCl or low glucose levels (A). Mating efficiencies of WT and Δ*tor1* strains transformed with the indicated plasmids. The results are the mean values of three independent experiments (B). ***C-E*,** Chromosomal expression of the *gad8-K263C* mutation suppresses growth and cellular defects in Δ*tor1* cells. Serial dilutions of cells plated onto rich medium (YES) medium at 30°C (no stress), 37°C, or at 30°C with 1M KCl (C) or low glucose levels (D). Mating efficiencies of the indicated strains. The results are the mean values of three separate experiments (E).

We next examined the ability of *gad8*-*T260C* or *gad8*-*K263C* to suppress additional defects associated with loss of TORC2. Cells disrupted for *tor1*^+^ are unable to grow under low-glucose conditions [41] and are severely defective in undergoing sexual development in response to starvation cues [34, 35], a process in which two opposite mating-type cells undergo conjugation to form the diploid zygote that subsequently undergoes meiosis and sporulation. We found that the *gad8*-*T260C* or *gad8*-*K263C* mutant alleles suppressed low-glucose stress sensitivity in Δ*tor1* cells (Fig 2A) and partially suppressed the inability of Δ*tor1* cells to undergo sexual development pathway (Fig 2B). Expression of *gad8*-*T260C* or *gad8*-*K263C* had no detectable deleterious effects in wild-type cells (Fig 2A). Disruption of any of the essential subunits of TORC2, *ste20*^+^ (Rictor in higher eukaryotes) or *sin1*^+^ (mSin1), also results in pleiotropic defects under stress conditions (S2A and S2B Fig). Similar to the suppression observed in Δ*tor1* cells, the *gad8*-*T260C* or *gad8*-*K263C* mutation suppressed the sensitivity of Δ*sin1* or Δ*ste20* cells to high temperature, osmotic stress or low-glucose (S2A and S2B Fig) and partially suppressed the sterility of Δ*ste20* cells (S2C Fig). Thus, *gad8*-*T260C* or *gad8*-*K263C* suppresses phenotypes associated with loss of TORC2 and is not specific for loss of Tor1.

We chose to further focus on the K263C mutation, since this mutation occurs in a highly conserved residue in the family of AGC kinases. We integrated the *gad8-K263C* allele into its natural chromosomal locus, under the native promoter of *gad8*^+^. The chromosomally integrated mutation suppressed the high temperature, osmotic stress or low-glucose sensitivities in Δ*tor1* cells (Fig 2C and 2D) and partially suppressed the mating deficiency in Δ*tor1* cells (Fig 2E).

### The kinase activity of Gad8-K263C is independent of TORC2

**Fig 3:**
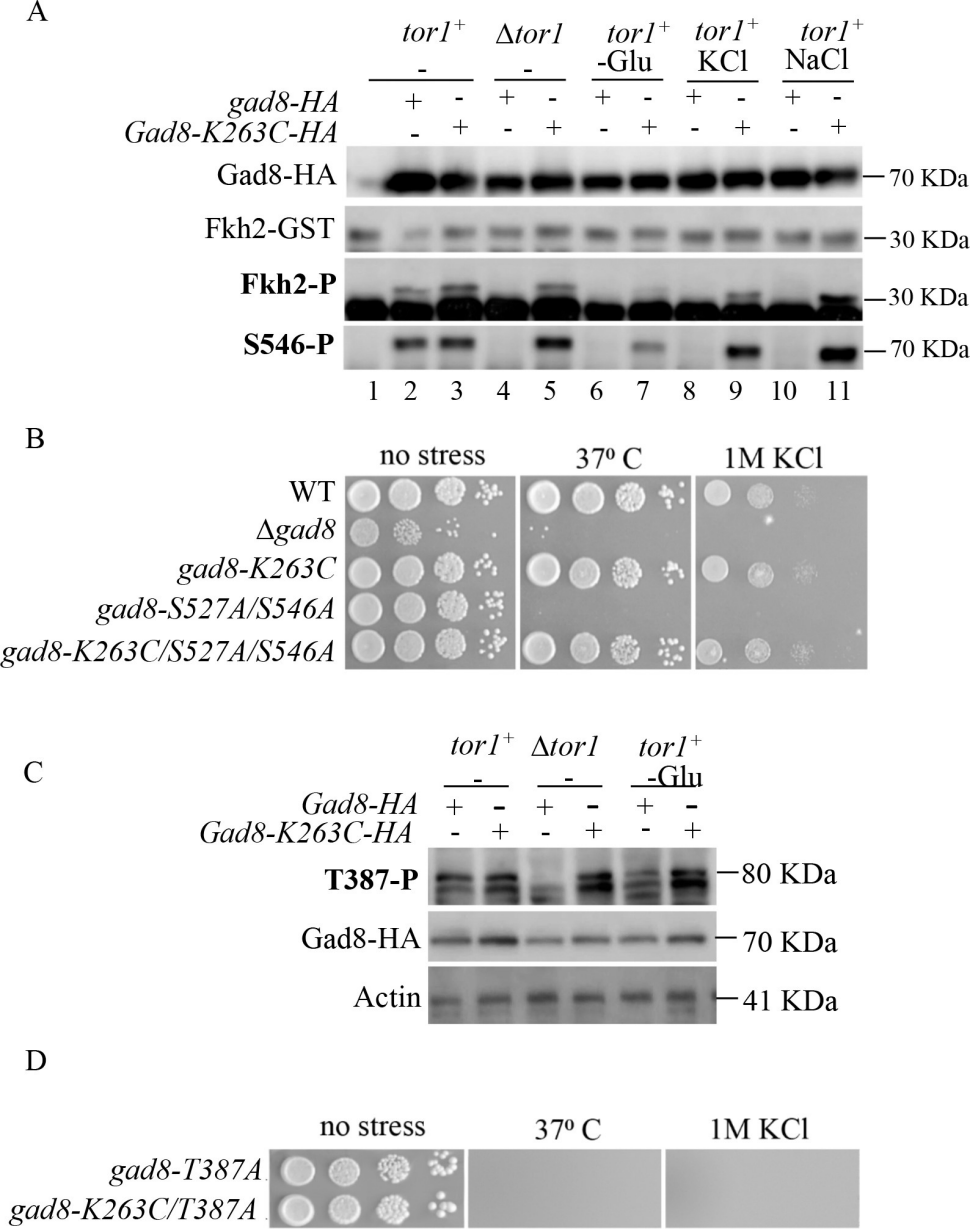
Gad8-K263C activity is independent of Tor1 *in vitro* and *in vivo*. ***A*,** Western blot analysis of protein extracts isolated from strains expressing no-tag Gad8 (lane 1), Gad8-HA or Gad8-K263C-HA (lanes 2–11). Cells were grown to mid-log phase and left untreated in YES medium or shifted for 1 h to EMM containing no carbon source (EMM-Glu) or to YES containing 1M KCl or 1 M NaCl. Gad8-HA and Gad8-K263C-HA were immunoprecipitated and assayed *in vitro* for their kinase activity using the Fkh2-GST peptide as a substrate. Phosphorylation of Fkh2-GST was detected with anti-PAS antibodies recognizing the phosphorylated form of the consensus sequence of AGC kinases (Fkh2-P). Phosphorylation of Gad8 at S456 was detected with anti-Gad8-S546-P phsophospecific antibodies (S546-P). ***B*,** Serial dilutions of the indicated strains spotted onto YES (no stress) or YES medium containing KCl and incubated at 30^0^ C or 37^0^ for 3 days. All mutations are chromosomal. ***C*,** Western blot analysis of protein extracts isolated from wild-type (*tor1*^+^) or Δ*tor1* cells grown in YES medium to mid-log phase or shifted for 1 hour into EMM containing no carbon source (-Glu). The status of phosphorylation of Gad8 at T387, the target site for Ksg1 (PDK1) was detected with anti-Gad8-T387-P phosphospecific antibodies. ***D*,** Serial dilutions of the indicated strains as described in B.

Tor1 phosphorylates Gad8 at S546 in the HM and at S527 in the TM, whereas Ksg1 phosphorylates Gad8 at T387 in the activation loop [33]. Previous studies have shown that the *in vitro* kinase activity of Gad8 is undetectable in Δ*tor1* cells [33, 37, 40]. Consistently, mutational analysis demonstrated that the phosphorylation events of Gad8 at the HM and TM by TORC2 are required for Gad8 activity *in vivo* [33]. To examine the kinase activity of Gad8-K263C, we used a nonradioactive *in vitro* kinase assay, which monitors the phosphorylation of Fkh2 by Gad8 [59]. We chromosomally tagged *gad8-K263C* with the 6HA epitope and compared its kinase activity to that of chromosomally expressed wild-type Gad8-6HA. In the presence of *tor1*^+^, both Gad8 and Gad8-K263C were active (Fig 3A, Fkh2-P, lanes 2–3), while only Gad8-K263C retained its kinase activity in the absence of Tor1 (Fig 3A, Fkh2-P, lanes 4–5). Thus, the suppression activities observed for *gad8*-*K263C* in cells lacking *tor1*^+^ correlate with Gad8-K263C kinase activity *in vitro*. Several laboratories, including us, have previously shown that Gad8 kinase activity is diminished in response to short exposure to osmotic stress or low glucose [40–42, 60]. Unlike Gad8, Gad8-K263C retained its kinase activity in response to 1-hour exposure to osmotic stress (1M KCl or NaCl, Fig 3A, Fkh2-P, lanes 8–11), further supporting Tor1-independent activation of Gad8-K263C. In response to glucose depletion, we detected a relatively weak phosphorylation of Fkh2 in *gad8-K263-6HA* cells (Fig 3A, Fkh2-P, lanes 7).

So far the only kinase that is known to phosphorylate Gad8 at S546 is Tor1. To our surprise, Gad8-K263C was phosphorylated at S546 in wild type cells, as well as in Δ*tor1* cells (S546-P, Fig 3A). This finding suggests that the Gad8-K263C mutant is phosphorylated by a kinase that normally does not recognize Gad8 as a substrate. Gad8-K263C was also phosphorylated at S546 under conditions that compromise Tor1 activity, such as osmotic or low glucose stress (S3A Fig), further supporting Tor1-independent phosphorylation of Gad8-K263C by an as yet unknown kinase. The only conditions under which we did not detect phosphorylation of Gad8-K263C were in Δ*tor1* cells in the presence of hydroxyurea or camptothecin (S3B Fig), a finding that may suggest that the activity of the kinase responsible for Gad8-K263C phosphorylation is inhibited under genotoxic stress conditions.

In order to examine whether Gad8-K263C requires the phosphorylation at TORC2-dependent sites for its activity *in vivo*, we combined TORC2-dependent non-phosphorylatable mutations in *gad8*, S546A and S527A, together with the K263C mutation. As previously described [33], mutating both Tor1-dependent phosphorylation sites, S546 and S527, to alanine, abolished the ability of cells to grow at high temperature or in the presence of osmotic stress (Fig 3B). In contrast, cells carrying the non-phosphorylatable mutations in *gad8* together with the K263C mutation, *gad8*-*K263C*/*S527A*/*S546A*, were able to grow at high temperature or in the presence of osmotic stress (Fig 3B). Thus, the K263C mutation reversed the temperature and osmotic stress sensitivities of *gad8*-*S527A*/*S546A*. We conclude that the phosphorylation at the TORC2-dependent sites is not required for the activity of the Gad8-K263C kinase.

Previous findings suggest a model in which Tor1-dependent phosphorylation of Gad8 on S546 facilitates the interaction with Ksg1 and enhances subsequent phosphorylation by Ksg1 at T387 [33]. This suggestion is primarily based on the finding that mutating S546 into aspartic acid mimicked the effect of phosphorylating Gad8 *in vivo* and induced a stronger activity of Ksg1 towards phosphorylation of Gad8 *in vitro* [33]. Using specific antibodies against the phosphorylated form of Gad8-T387, we found that Gad8-K263C is phosphorylated at T387 in Δ*tor1* cells under normal or low-glucose growth conditions (Fig 3C). The wild type Gad8 is not phosphorylated at T387 in the absence of Tor1 and is only weakly phosphorylated under low-glucose conditions (Fig 3C). This finding suggests that the K263C mutation allows Ksg1 to interact with and to phosphorylate Gad8 in the absence of Tor1. In order to check whether phosphorylation of T387 by Ksg1 is required for Gad8-K263C activity *in vivo*, we introduced the T387A mutation into the chromosomal *gad8* locus, either alone or in combination with the K263C mutation. As previously described, *gad8-T387A* cells failed to grow under stress conditions [33]. Strains carrying both the T387A and K263C mutations, *gad8-T387A/K263C*, also failed to grow under stress conditions (Fig 3D), indicating that Gad8-K263C still requires activation by Ksg1.

### The *gad8-K263C* mutation is located at the PIF pocket and induces higher flexibility at Gad8-T387, the PDK1-dependent phosphorylation site

**Fig 4:**
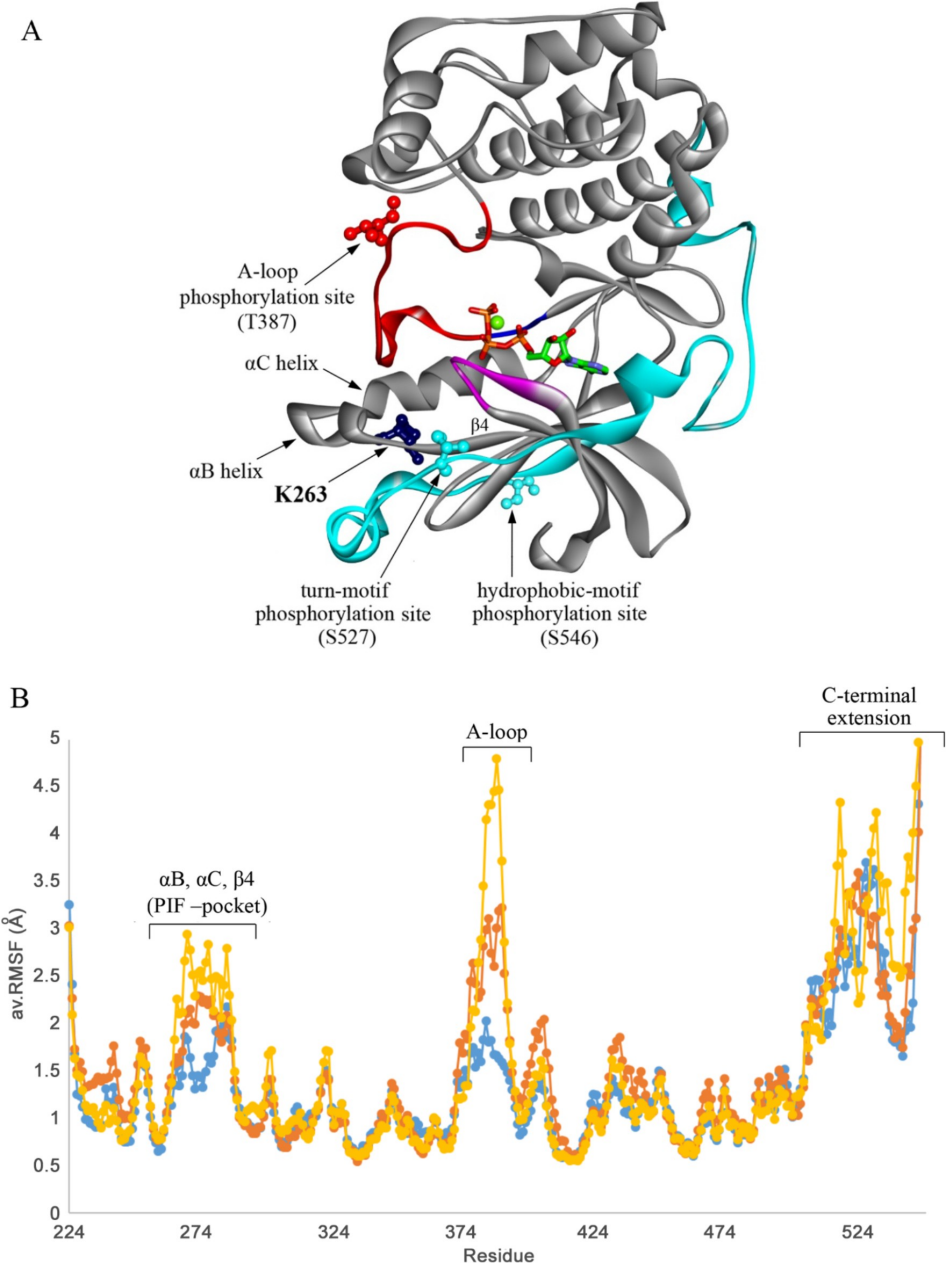
The *gad8-K263C* mutation is located at the PIF pocket and induces higher flexibility at the PIF pocket and activation loop *A*, 3D model of *S*. *pombe* Gad8 structure. The protein is shown as a ribbon diagram (grey), the ATP is shown as sticks, and the Mg ion is shown as a green sphere (ATP is colored according to atom types). The activation loop (A-loop) is shown in red. The highly conserved structures in AGC kinases, the DFG-motif and the Gly-rich loop are colored in blue and magenta, respectively. The C-terminal extension in cyan. The residues K263, T387, S527, and S546 are shown in balls and sticks. ***B*,** RMSF plot of the backbone atoms for non-phosphorylated Gad8 (blue), Gad8-pS527/pS546 (yellow, p denotes phosphorylation), and non-phosphorylated K263C-Gad8 (orange).

**Fig 5:**
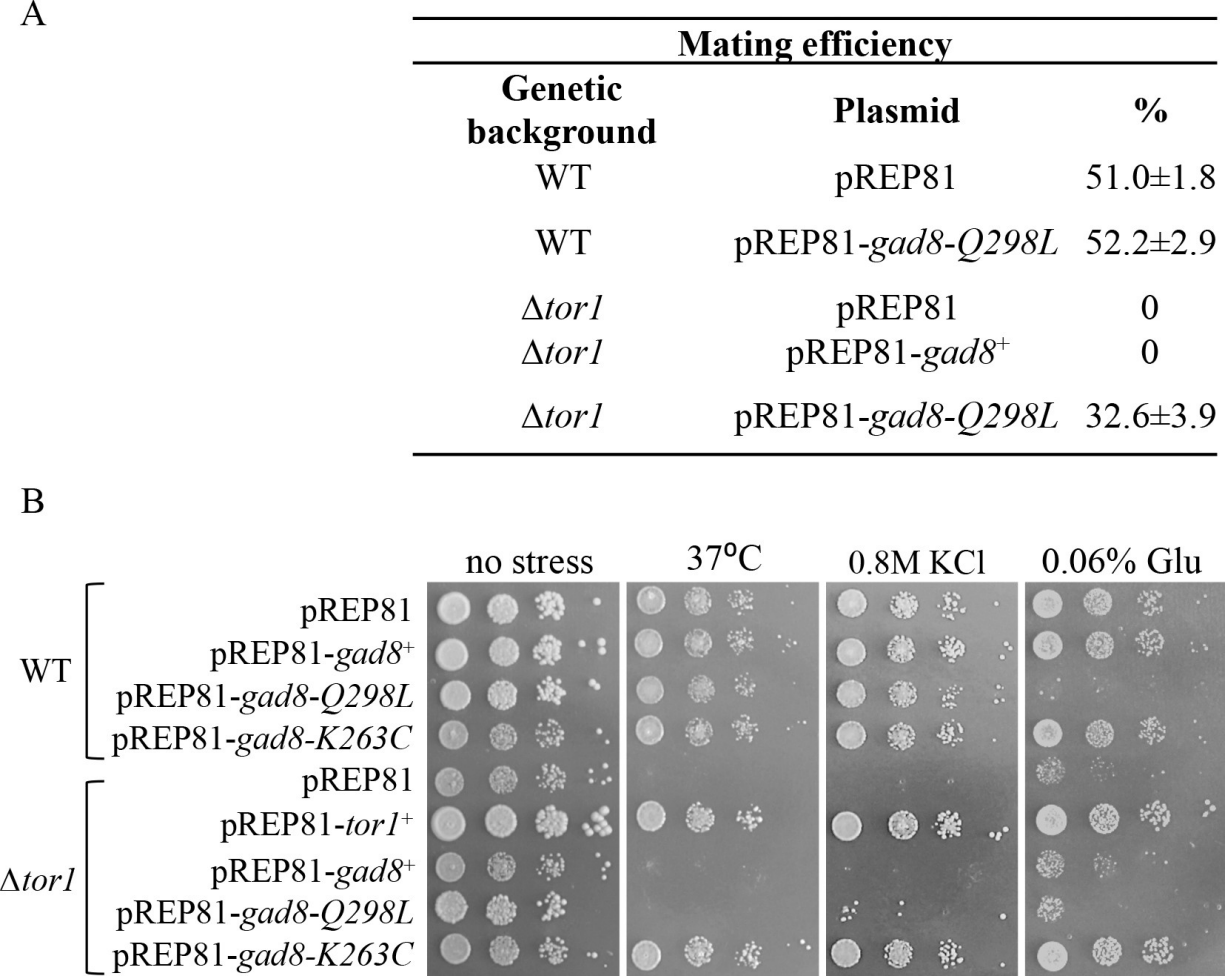
*gad8-Q298L* partially suppresses the mating type defect, but not osmotic stress, high temperature or low-glucose stress sensitivities in Δ*tor1* cells *A*, Mating efficiencies of the indicated strains. The results are the mean values of three separate experiments. ***B*,** Serial dilutions of cells plated onto minimal medium containing no stress, 1M KCl, or reduced glucose levels. Plates were incubated at 30°C, unless otherwise indicated.

In the absence of a three-dimensional X-ray structure of *S*. *pombe* Gad8, a ligand-supported homology model of Gad8 in its un-phosphorylated state was generated using its homologue SGK1 kinase (PDB code: 2R5T, 47% sequence identity). The αC-helix and the C-terminal extension regions were modeled based on AKT1 and PKCα kinases (PDB codes: 6NPZ and 4RA4, respectively). The final model is presented in Fig 4A. The model was shown to have high quality stereochemical profile (94% of the amino acids in the core regions of the Ramachandran plot). The general 3D organization of Gad8 comprises an N-lobe domain of beta sheets, and a C-lobe domain mainly consisting of alpha-helices and loops. The two lobes are connected to each other by a hinge region that defines the activation loop. At the interface of the two domains there is a deep hydrophobic cleft that binds ATP and a Mg^2+^ ion. The point mutation K263C is located at the small N-lobe between the αB-helix and the β4 beta-sheet, that together with the αC helix delimit the PIF-pocket. As described above, Gad8-K263C bypasses the need for phosphorylation by Tor1 at the TM and HM motifs, S527 and S546, respectively. However, there is still a need for a phosphorylation by Ksg1 at the activation loop (Thr387) to get a full activation of Gad8. Therefore, we speculated that the mutation K263C may alter the protein structure in a manner similar to that affected by Tor1 phosphorylation. In order to get insights into the effect of the mutation on the protein structure, three constructs, namely un-phosphorylated Gad8, Gad8-K263C, and Gad8 phosphorylated at both the HM and TM (Gad8-pS527/pS546) were subjected to molecular dynamics (MD) simulations. Each construct was simulated three times for 50 ns. Each replica was started from a different random seed. The stability of the resulted trajectories was tested based on the root mean square deviation (RMSD) of the backbone atoms with respect to the equilibrated structures. Next, the root mean square fluctuation (RMSF) was calculated in order to determine the degree of flexibility of each residue in each construct. RMSF plots of protein backbone atoms of un-phosphorylated Gad8, Gad8-K263C and Gad8-pS527/pS546 during the simulations are shown in Fig 4B. It is evident from this analysis that the activation loop in Gad8-K263C and Gad8-pS527/pS546 is more flexible than in un-phosphorylated Gad8. Furthermore, the PIF-pocket region shows higher flexibility in Gad8-K263C and Gad8- pS527/pS546 compared to un-phosphorylated Gad8. We have identified an H-bond interaction between K263 and Q298 in WT-Gad8, which occurs in ~62% of the simulation time. Interestingly, this H-bond interaction appears in Gad8-pS527/pS546 only in ~22% of the simulation time, and does not appear at all in Gad8-K263C as a result of the point mutation. This may explain the higher flexibility of the PIF-pocket region in Gad8-K263C and Gad8-pS527/pS546. The Q298 residue is located at the β4 sheet in the PIF pocket and is also highly conserved in evolution (S4 Fig). Mutating the Q298 residue into leucine, *gad8-Q298L*, partially suppressed the mating deficiency of Δ*tor1* cells when expressed from the pREP81 plasmid (Fig 5A), suggesting that the MD analysis is useful in predicting additional *gad8* alleles that bypass the requirement for TORC2. The *gad8-Q298L* did not suppress osmotic, high temperature or low glucose sensitivities in Δ*tor1* cells (Fig 5B). Moreover, over-expression of *gad8-Q298L* in wild type cells showed sensitivity to low-glucose conditions (Fig 5B). It is possible that mutation at Q298 interfere with substrate(s) recognition motif or docking interaction with substrate(s) that are specifically required for viability under low glucose conditions. Possible reasons for the differential suppression activities in *gad8* mutant alleles are further discussed below.

### *gad8-K263C* or *gad8-T260C*, but not *gad8*-*Q298L* cells, are sensitive to DNA damage or DNA replication stress

**Fig 6:**
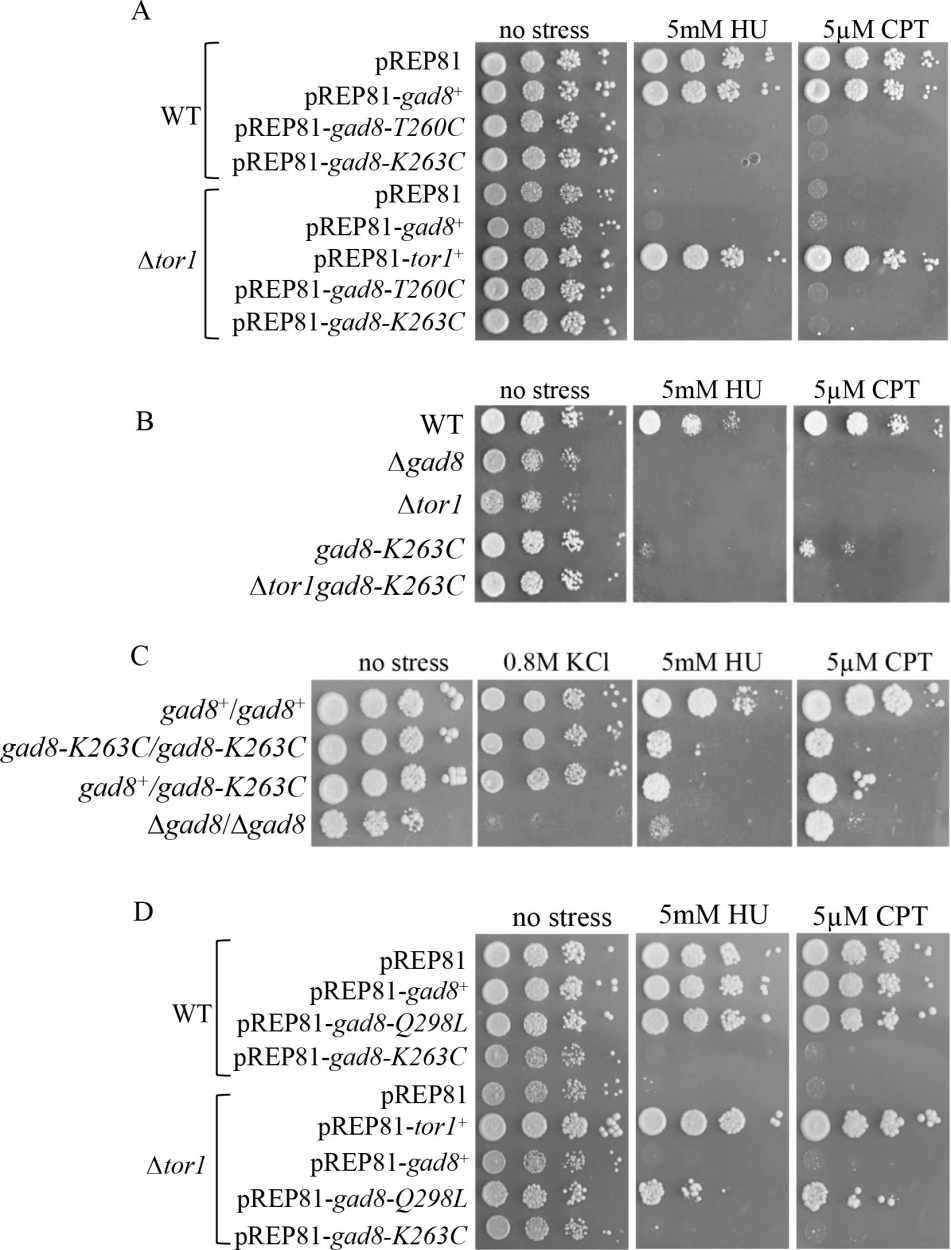
*gad8-K263C* or *gad8-T260* confer sensitivity to DNA damaging conditions. ***A*,** Plasmids containing *gad8-K263C* or *gad8-T260* confer genotoxic stress in wild-type cells and are unable to suppress genotoxic sensitivity in Δ*tor1* mutant cells. WT or Δ*tor1* cells transformed with empty vector (pREP81), or with vector carrying *tor1*^+^, *gad8*^+^, *gad8-T260C* or *gad8-K263C* were serially diluted and spotted onto minimal medium with no stress or containing 5mM HU or 5μM CPT. Plates were incubated at 30^0^ C for 3 days. ***B*,** The chromosomal K263C mutation confers sensitivity to genotoxic stress. WT, Δ*tor1*, *Δgad8*, *gad8*-*K263C* or Δ*tor1gad8*-*K263C* cells were serially diluted and spotted onto minimal medium with no stress or containing 5mM HU or 5μM CPT and incubated at 30^0^ C for 3 days. Cells spotted on 0.8M KCl are used as control. ***C*,** *gad8-K263C* confers dominant-negative effect under genotoxic stress conditions. Serial dilutions of diploid cells were spotted onto the indicated plates. Plates were incubated at 30^0^ for 3 days. ***D*,** The *gad8-Q298L* partially suppresses genotoxic stress sensitivity in Δ*tor1* cells. Serial dilutions of WT or Δ*tor1* cells harboring the indicated plasmids were spotted onto minimal medium or minimal medium containing 5mM HU or 5μM CPT and incubated at the indicated temperatures for 4 days.

We have previously shown that cells lacking TORC2 or *gad8*^+^ are highly sensitive to DNA replication stress or DNA-damaging conditions [39, 56]. We employed the kinase-dead *gad8* allele, *gad8-K259R* [33] to examine whether the kinase activity of Gad8 is required under these conditions. Cells carrying *gad8-K259R* are sensitive to genotoxic stresses, indicating that the kinase activity of Gad8 is required under these conditions (S5A Fig). Additionally, the phosphorylation sites of Gad8 are required for genotoxic stress, since *gad8-S527A/S546A* mutant alleles are also sensitive to DNA damage and DNA replication stress (S5B Fig). We therefore examined the ability of pREP81-*gad8*-*K263C* or pREP81-*gad8*-*T260C* to suppress the sensitivity of TORC2 mutant cells to genotoxins. We first examined the ability of *gad8*-*K263C* or *gad8*-*T260C* to suppress the sensitivity of Δ*tor1*, Δ*ste20* or Δ*sin1* to hydroxyurea (HU) or camptothecin (CPT). HU generates replication stress by depleting the dNTP pools, while CPT stabilizes covalent DNA topoisomerase-I complexes, leading to collision of DNA replication forks with the drug-enzyme-DNA complex and inducing double strand breaks [61]. *gad8-K263C* or *gad8-T260C* expressed from the pREP81 plasmid failed to suppress the sensitivity of Δ*tor1* (Fig 6A) or the sensitivity of Δ*ste20 or* Δ*sin1* to HU or CPT (S6A Fig). Moreover, *gad8-K263C* or *gad8-T260C* expressed from the pREP81 plasmid in wild-type cells conferred sensitivity to HU or CPT (Fig 6A). The chromosomally integrated *gad8-K263C* mutation, in an otherwise wild-type background, also conferred a strong sensitivity to HU or to CPT (Fig 6B). Compared with Δ*tor1* or Δ*tor1 gad8-K263C* cells, the chromosomally integrated *gad8-K263C* mutation exhibited a slightly less severe genotoxic stress sensitivity (Fig 6B), which may suggest that *gad8-K263C* is not fully impaired under genotoxic stress conditions or that Tor1 also acts independently of Gad8 in promoting genotoxic resistance. Notably, heterozygous *gad8*^*+*^*/gad8-K263C* diploid cells are as sensitive to HU or CPT as *gad8-K263C/gad8-K263C* cells, suggesting that the K263C mutation acts in a dominant-negative manner with respect to genotoxic stress sensitivity (Fig 6C). Mutating the K263 into alanine or arginine (negativelly charged residues, similar to lysine), K263A or K263R, resulted in suppression of the high temperature or high osmolarity sensitivity of Δ*tor1* cells, but conferred genotoxic stress sensitivity in wild-type cells (S6B Fig). Thus, K263 itself appears to be crucial for genotoxic stress responses.

Unlike the K263C or T260C mutations, the Q298L mutation did not impair the ability of Gad8 to survive genotoxic stress conditions and showed a partial ability to suppress the sensitivity of Δ*tor1* cells to HU or CPT (Fig 6D). We also found that the *gad8-S527D/S546D* allele, containing aspartic acid residues at the phosphorylation sites of TORC2 [33] suppressed the sensitivity to high temperature and osmotic stress, as well as the sensitivity to CPT or HU (S6C Fig). Thus, constitutive activation of Gad8 in itself does not confer sensitivity to either of the stress conditions examined. We conclude that mutations in the PIF pocket of Gad8 bypasss the requirement for TORC2 under specific sets of stress conditions, but depending on the specific alteration, these muations may also impair the protein function under other stress conditoins.

### Under genotoxic stress conditions, the *gad8-K263C* mutation confers similar phenotypes to loss of function of *gad8*^+^

**Fig 7:**
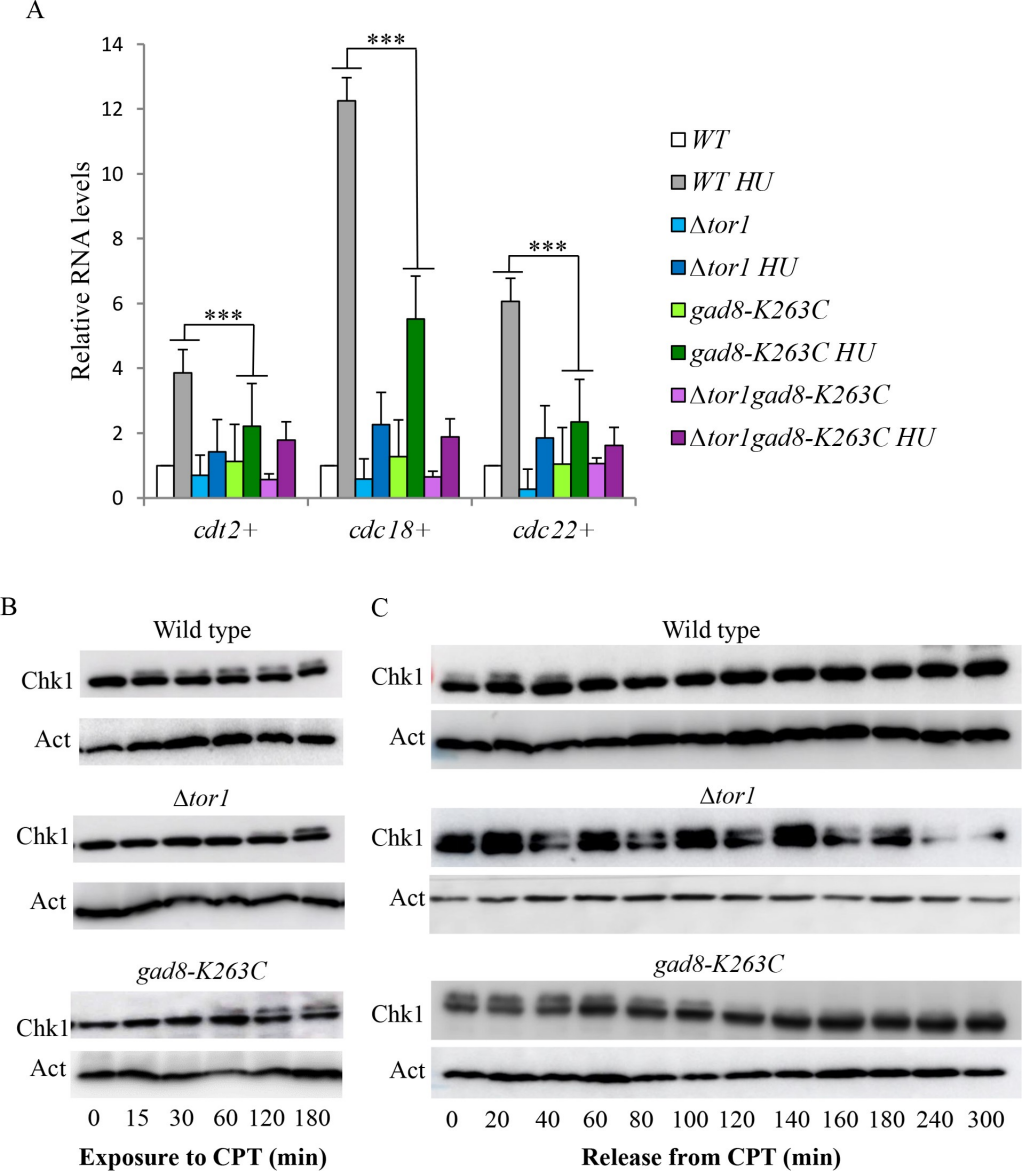
*gad8-K263C* cells show similar defects to Δ*tor1* cells with respect to DNA damage responses *A*, Expression levels of *cdt2*^+^, *cdc18*^+^ and *cdc22*^+^ in WT, Δ*tor1*, *gad8-K263C* and Δ*tor1gad8-K263C* cells were determined by qRT-PCR. Total RNA was prepared from untreated cells or cells treated with 12mM HU for 3 h. The level of *act1*^+^ mRNA was used as a reference. Error bars (SD) were calculated from biological triplets and significant differences were determined by the Student's *t*-test (*** *P* < 0.001). ***B*,** Chk1 phosphorylation is delayed in *gad8-K263C* in response to CPT. Wild-type, Δ*tor1* or *gad8-K263C* cells containing an HA-tagged Chk1 were grown to mid-log phase and treated with 30 μm CPT. Protein extracts from each of the indicated time points were analyzed by Western blotting. Membranes were probed with anti-HA to visualize Chk1 and with anti-Actin as a loading control. The upper band observed with anti-HA antibodies represent the phosphorylated and activated band of Chk1. ***C*,** De-phosphorylation of Chk1 is delayed in *gad8-K263C* mutant. Cells were arrested for 3 h in 30μM CPT before being released into fresh medium. Protein samples from the indicated time points were analyzed by Western blot as describe above.

Dominant mutations often exhibit a phenotype that is different from that of loss of function of a given gene. Therefore, the finding that *gad8-K263C* cells shows similar defects to Δ*tor1* or Δ*gad8* cells with respect to genotoxic sensitivity is somewhat surprising. We further explored possible underlying mechanisms for the sensitivity of *gad8-K263C* mutant cells to genotoxins. Under DNA replication stress, *S*. *pombe* cells induce a group of ~20 genes that are under the transcriptional regulation of the MluI cell cycle box-binding factor (MBF) complex [62]. We have previously shown that in mutant cells lacking TORC2 or Gad8, the induction of the MBF target genes in response to replication stress is significantly reduced compared with wild-type cells [63]. Examination of the level of induction of three representative MBF target genes, *cdc22*^+^, *cdc18*^+^, *cdt2*^+^, revealed that these are also poorly induced in response to replication stress (HU) in *gad8-K263C*, or Δ*tor1 gad8-K263C* mutant strains (Fig 7A). For the *cdc18*^+^ gene, the level of induction in *gad8-K263C* cells is not as impaired as in cells carrying Δ*tor1*, yet, they are significantly reduced compared with wild-type cells (see *p* values and compare grey with green bars in Fig 7A).

Exposure of cells to DNA damage leads to the activation of the main DNA damage checkpoint kinase, Rad3 (ATR), resulting in the activation of the downstream effector kinases Chk1 or Cds1 [61]. In response to CPT, Chk1 becomes activated by phosphorylation, which is detectable as a slow migrating band in Western blot analyses, and is essential for propagating the checkpoint activation signal [64]. As previously reported, Δ*tor1* mutant cells show a delay in the phosphorylation of Chk1 in response to CPT compared with wild-type cells ([39] and see Fig 7B). Here we show that *gad8-K263C* cells showed an intermediate defective phenotype with respect to phosphorylation of Chk1 in response to CPT. Thus, while wild-type and Δ*tor1* cells show phosphorylation of Chk1 15 and 120 minutes following exposure to CPT, respectively; *gad8-K263C* cells start showing phosphorylation of Chk1 60 minutes following exposure to CPT (Fig 7B).

Despite the delayed phosphorylation of Chk1, Δ*tor1* or Δ*gad8* arrest cell cycle progression in response to CPT treatment and survive short exposures to the DNA damaging conditions [39]. In contrast, continuous exposure to CPT and other DNA damages conditions is detrimental to TORC2 mutant cells [39]. This differential sensitivity to short versus continuous exposure to DNA damage suggests that TORC2 mutant cells are defective in later responses to DNA damage, such as DNA damage repair and/or re-entrance into the cells cycle. Consistently, there is a marked delay in de-phosphorylation of Chk1 in Δ*tor1* cells following removal of the DNA damage [39]. Again, *gad8-K263C* cells showed an intermediate level of defect in de-phosphorylation of Chk1 following damage removal: Chk1 becomes de-phosphorylated 120 minutes following removal of CPT in *gad8-K263C* cells, compared to 60 minutes in wild-type cells and 240 minutes in Δ*tor1* cells (Fig 7C). In conclusion, under genotoxic stress conditions, despite its dominant nature, the *gad8-K263C* mutation shows a similar, albeit less severe phenotype to loss of function of *gad8*^+^.

## Discussion

TOR (Target of Rapamycin), which can be found in two structurally and functionally distinct complexes, is a major regulator of growth, proliferation and survival [1–3]. While initially TORC1 has been the focus of the majority of studies of TOR signaling, the recent decade has seen a growing interest in TORC2. This has been fueled by the findings that dysregulation of mTORC2 plays an important role in several human diseases [1]. TORC2 in *S*. *pombe* mediates the phosphorylation and activation of the downstream AGC-family protein kinase Gad8, which is orthologous to AKT1 and SGK1 [33]. Here, we report the isolation of *gad8* mutant alleles that bypass the requirement for TORC2-mediated phosphorylation under distinct sets of physiological conditions. These mutations map into the PIF pocket of Gad8, a conserved hydrophobic pocket in AGC kinases, which regulates both inter- and intra-molecular interactions that affect ATP-binding and kinase activity and has been shown to specifically affect the docking of PDK1 to its downstream AGC kinases [45, 46]. We isolated the *gad8-T260C* and *gad8-K263C* mutations by a genetic screen designed to identify Gad8 mutant proteins that do not require TORC2 for growth under high temperature conditions. The *gad8-T260C* and *gad8-K263C* mutant alleles, as well as *gad8-K263A* or *gad8-K263R*, suppress the inability of TORC2 mutant cells to grow under high temperature, osmotic stress or low-glucose conditions and partially suppress the requirement for TORC2 for sexual development. *gad8-T260C*, *gad8-K263C*, *gad8-K263A* or *gad8-K263R* did not suppress the sensitivity of TORC2 mutant cells to DNA replication stress or DNA damaging conditions. Indeed, these mutations confer sensitivity to genotoxic stresses when expressed in wild-type cells, demonstrating a dominant-negative effect in heterozygous diploid cells. Cells that chromosomally express the *gad8-K263* mutation show a phenotype similar to the one conferred by loss of function of TORC2 with respect to genotoxic stress sensitivity, including low levels of MBF-gene induction and defects in both activation and inactivation of the DNA damage checkpoint. In future experiments it will be interesting to explore the possibility that mutations at K263 interfere with substrate(s) recognition motif or docking interaction with substrate(s) that are specifically required for the genotoxic stress response. The observed dominant negative phenotype could result from higher affinity of a defective Gad8 mutant protein to its substrate. Further studies identifying the substrates of Gad8 are required to support such speculations.

Based on our MD analyses, we introduced another mutation in the PIF pocket of Gad8, Q298L. The Q298L mutation could partially suppress the mating deficiency and genotoxic stress sensitivities of cells lacking TORC2, but did not suppress the high temperature or osmotic sensitivities. The Q298L appears to confer a dominant negative effect under condition of low glucose. These findings suggest that while mutations in the PIF pocket can render Gad8 independent of TORC2, they may also impair the protein function under specific stress conditions, possibly by interfering with interactions with specific substrates.

Similar to other AGC kinases, Gad8 requires at least three types of phosphorylation events; by TORC2 at its hydrophobic (HM, S546) and turn-motif (TM, S527) and by the PDK1-like protein, Ksg1, at its activation loop (T387) [33]. We demonstrate that the *in vitro* kinase activity of Gad8-K263C is maintained in the absence of Tor1 and that combining the K263C mutation together with mutations in the phosphorylation sites of TORC2, S527A and S546A results in a mutant allele, *gad8*-*K263C*/*S527A*/*S546A*, that is able to grow at high temperature or in the presence of osmotic stress. In contrast, combining the K263C mutation with mutations in the phosphorylation sites of Ksg1 results in a mutant allele that is sensitive to these stresses. Thus, our genetic and biochemical analyses suggest that the K263C mutation bypass the requirement for TORC2-dependent phosphorylation at the HM and TM, but not the phosphorylation at the activation loop by Ksg1. How, then, does the mutation K263C mutation in the PIF pocket of Gad8 bypass the requirement for TORC2? Our MD studies suggest that the K263C mutation causes a higher flexibility of the activation loop and the PIF region. The higher flexibility of the activation loop may allow it to explore wider conformational space, thus facilitating the phosphorylation of Thr387 by Ksg1. MD analysis of Gad8 phosphorylated at both the HM and TM similarly resulted in higher flexibility at the PIF domain and the activation loop, further suggesting an intramolecular activation path that involves the C-terminal phosphorylation sites, the PIF domain and the activation loop. Additionally, our MD analysis indicated the presence of an H-bond between K263 and Q298 in the predicted non-phosphorylated structure of Gad8. This H-bond may contribute to the rigidity of the PIF pocket. Mutating the Q298 residue into leucine resulted in partial suppression of the sexual development defect or genotoxic stress sensitivity of Δ*tor1* cells, suggesting that Q298 is important to render Gad8 activity dependent on TORC2. Our ability to predict mutations that bypass the requirement of TORC2 under stress conditions supports the validity of our MD analyses and calls for future studies of the role of the PIF pocket in rendering Gad8 dependent on activation by TORC2. Since the PIF pocket and the mode of activation of AGC kinases by TOR complexes are both highly conserved in evolution, similar mutations in AGC kinases may also result in TORC2-independet activities.

The PIF pocket residues K263 and Q298 are highly conserved in the family of AGC kinases. The SGK1-K131 residue, which is equivalent to K263, was suggested to play a role in localization of SGK1 as part of a nuclear localization sequence (NLS) [65]. However, the NLS in SGK1 conforms to the consensus bipartite NLS (KKAILKKKEEK), while the equivalent Gad8 sequences does not (KKAHIVSRSEV). Analysis of the equivalent residues in PDK1 and AKT suggested that when the HM is phosphorylated, the equivalents of K263 and Q298 in PDK1 and AKT bind the first two phenylalanine residues in the HM [49, 52]. Mutations analyses of PDK1-K115 indicated that K115 is important for PDK1 binding to artificial PIF peptides [49] and is critical for binding and phosphorylation of the PDK1 target kinases, S6K, RSK SGK, but not other substrates [51]. Thus, PDK1-K115 is important for docking to substrate AGC kinases but does not significantly alter the conformation of the active site of PDK1 [49, 51]. PDK1 is different compared with other AGC kinases as it interacts with and phosphorylates, downstream AGC kinases. The role of the PIF pocket in other AGC kinases has been less-extensively studied. Our studies expand our understanding of the PIF pocket and suggest that it plays a role in keeping Gad8, and possibly other AGC kinases, dependent on TORC2 activation.

## Materials and methods

### Yeast strains, media and growth assays

*S*. *pombe* strains used in this paper are listed in S1 Table. Yeast cells were cultured in rich YES medium supplemented with adenine and uracil or in Edinburgh minimal medium (EMM, 5 g/liter NH_4_Cl), as described in [66, 67]. EMM was supplemented with amino acids according to the auxotrophic requirements of the strains used. EMM-G is EMM containing no glucose. For cell growth assays, logarithmic growing cells were either streaked or serially diluted and spotted on solid YES or EMM plates. Stress sensitivity of *S*. *pombe* cells was assessed by streaking or spotting on YES or EMM plates supplemented with KCl, hydroxyurea (HU) or camptothecin (CPT), or on plates with reduced levels of glucose, or plates that are incubated at a relatively high temperature (37°C). Strains that contained plasmids were assessed on EMM plates in order to select for the plasmid, while strains that contained chromosomal integrations were assessed on YES plates. Unless otherwise specified, plates were incubated at 30° for 4 days before being photographed.

### Genetic screens for the isolation of Tor1-independent *gad8* mutant alleles

We obtained *gad8* mutant alleles by using random PCR-based mutagenesis. 5 ng of pREP81-*gad8*^+^ plasmid was taken for PCR amplification of the *gad8*^+^ ORF with primers: #1324 (5'TCAATTGAATAAGTTGAATTAATTATTTCAATCTCATTCTCACTTTCTGACTTATAGTCGCTTTGTTAAATCATATGTCCTGGAAACTTACAAAGAGTATGTATTCCATA) and #1282 (5'ATAGTTTGAAAGAAAAACCCTAGCAGTACTGGCAAGGGAGACATTCCTTTTACCCGGGGATCCGCTAGCCCATGGGTCGATCACCTAATGACACTTCCAGGTGCT). The underlined sequences are homologous to pREP81 sequences and the rest of the primer is homologous to *gad8*^+^ sequences. Four separate PCR reactions were performed, and in each reaction the concentration of a different dNTP was decreased from the standard 2mM concentration to 2μM. Using Phire Hot Start II DNA Polymerase (ThermoFisher #F-122L), four cycles of PCR were performed with the following temperature profile: 94°C 1 min, 54°C 1 min, 72°C 2 min. The PCR products of the four separate PCR reactions were mixed together and 34 cycles of PCR were performed with the following temperature profile: 94°C 1 min, 48°C 2 min, 72°C 2 min. The resulting PCR fragments were co-transformed together with the pREP81 plasmid into *tor1*-L2045D cells (TA3110) using the lithium acetate procedure. Transformant cells were incubated at 37°C for 6 days. Approximately 23,000 colonies were screened. Plasmid DNA was isolated from cells that grew at 37°C and then used to transform bacterial cells for plasmid amplification. Plasmid DNA extracted from bacteria was retransformed into *tor1*-L2045D (TA3110) cells and into Δ*tor1* (TA1291) cells. Plasmids that suppressed the temperature sensitive phenotype of Δ*tor1* and *tor1*-L2045D cells were subjected to DNA sequence analysis.

### Chromosomal integration of the K263C mutation into *gad8*^+^

We integrated the K263C mutation into the chromosomal locus of *gad8*^+^ using standard techniques based on homologous recombination. We first obtained a fragment containing the K263 residue using wild-type genomics DNA as template for two separate PCR reactions. One reaction used primer #1388 (5'TATCTATGCTTTAAAAACTATGAAA**TGC**GCCCACATTGTATCTCGCAGTGAA) together with primer #252 (5'TCC CCCGGG TCACCTAATGACACTTCCAGG) and a second reaction using primer.

#251 (5' GGAATTC CAT ATGTCCTGGAAACTTACAAAGAG) together with

#1389 (5' CTTCACTGCGAGATACAATGTGGGC**GCA**TTTCATAGTTTTTAAAGCATAGATA). The underlined sequences indicate the site encoding for K263C. For the PCR reaction, 34 cycles of PCR were performed with the following temperature profile: 94°C 1 min, 54°C 1 min, 72°C 2 min. The resulting two PCR fragments were stitched together using 1μl of PCR product from each PCR reaction as template and primers #251 and #252. PCR amplification was achieved by 34 cycles of PCR with the following temperature profile: 94°C 1 min, 48°C 1 min, 72°C 2 min. The PCR product was transformed into the *gad8*::*ura*^+^ (TA1029) strain and plated onto 5-FOA plates for selection against *ura4*^+^ cells. Genomic DNA was isolated from cells that grew in the presence of 5-FOA and were further subjected to DNA sequence analysis to ensure the presence of the K263C mutation at the correct locus.

### Construction of *gad8* mutant alleles

The *gad8-K263A*, *gad8-K263R* and *gad8-Q298L* mutant alleles were constructed by site-directed mutagenesis using pREP81-*gad8*^+^ as the template and a pair of phosphorylated synthetic oligonucleotide primers (see S S2 Table) in a single-step plasmid amplification method, employing ALLin Mega HiFi Red Mastermix, 2X (HighQu # HLM0301). The amplified product was circularized by a ligation step and transformed into an *Escherichia coli* host. The resulting construct was confirmed by nucleotide sequencing.

### Assays for mating efficiency

Cells were grown at 30°C in EMM with adequate amino acid supplements to a density of approximately 5x10^6^ cells/ml. Cells were then spotted on EMM-N plates and incubated for three days at 25°C. An aliquot was taken for inspection under a light microscope and the numbers of cells, zygotes, and spores were counted. The percentage of mating was calculated by dividing the number of zygotes, asci, and free spores by the number of total cells. One zygote or one ascus was counted as two cells, and one spore was counted as a half cell. In each experiment 500 to 1,000 cells were counted.

### Protein extraction and immunoprecipitation (IP) assays

Cells were grown to mid-logarithmic phase, washed once with water, and resuspended in lysis buffer (20 mm Tris-HCl, pH 7.5, 0.5 mM EGTA, 0.5 mM EDTA, 1 mM DTT, 125 mM potassium acetate, 12.5% glycerol, 0.1% Triton X-100, protease inhibitor mixture, and 1 mM phenylmethylsulfonyl fluoride). Cells were broken for 45 min with glass beads, centrifuged for 10 min at 10,000 × *g* and the supernatant was collected. 20 μg of total protein extract was resolved on SDS-PAGE using 10% acrylamide gels. For immunoprecipitation, 1,000 μg of proteins were prepared and pre-cleared with 20 μl of protein A-Sepharose and protein G-Sepharose beads mixture (GE Healthcare). 2 μl of hemagglutinin (HA) antibodies were added to the cleared extract and incubated overnight at 4 ^0^C. The beads were washed once with lysis buffer, once with lysis buffer containing 0.5 M NaCl, and twice with buffer A (50 mm Tris-HCl, pH 7.5, 0.1 mm EGTA, 0.1% β-mercaptoethanol). The resulting immunoprecipitates were used for *in vitro* kinase assays.

### *In vitro* kinase assays

We applied a nonradioactive *in vitro* kinase assay for Gad8 [59], based on the use of GST-Fkh2 as a substrate [37]. For the Gad8 kinase assay, a DNA fragment encoding amino acid residues 291 (Gln) to 411 (Pro) of Fkh2 was expressed in *Escherichia coli* BL21 strain as GST fusion, using the pGEX-4T1 expression vector and purified. Cells expressing Gad8-HA extracts were immunoprecipitated, and the resultant immunocomplexes were resuspended in 30 μl of kinase buffer (10 mM MgAc, 100 mM ATP, and phosphatase inhibitor mixture, Sigma) containing 0.1 μg of GST-Fkh2. After incubation for 10 min at 30°C, the reaction was terminated by addition of 7 μl of 5 × SDS-PAGE sample buffers and incubated for 5 min at 80°C. The reaction was detected by Western blot analysis using anti-phospho-AKT substrate antibody (Cell Signaling Technology).

### Western blotting

Proteins were resolved by SDS-PAGE 10–15% acrylamide gels and transferred to nitrocellulose membranes, blocked with 5% milk in TBST and immunoblotted with the indicated antibodies. Detection was carried out using the ECL SuperSignal detection system (Thermo Scientific). Gad8 Thr387 phosphorylation events were detected using total protein extracts by phosphospecific antibodies. Antibodies against Gad8 Thr387-P were raised against the Gad8 phosphopeptide CRFANWpSYQRPT [59]. To follow Chk1 phosphorylation, proteins were extracted with trichloroacetic acid (TCA) as described previously and resolved by SDS-PAGE using 8% acrylamide gels.

### Real Time Quantitative PCR (qRT-PCR)

RNA extractions and qRT-PCR analysis were performed as described in [59]. 50 ml of each strain were grown to an A600 of ≈1 in minimal medium. RNA was prepared using the hot phenol method and treated with RNase free RQ1 DNase I (Promega) to remove DNA prior to reverse transcription. 1μg of RNA was reverse transcribed with ImProm-II Reverse Transcriptase (Promega), followed by Real-time PCR with Precision Fast qPCR Master mix kit (Primer Design). Reactions were performed in triplicates and run on the Step One Plus Real-Time PCR (Applied Biosystems). Threshold cycle (Ct) values for the cDNA of interest were normalized to the Ct values of *act1*^*+*^ and relative expression levels were quantified using the comparative method and calculated as 2^−ΔΔCt^. The amount of expression was expressed relative to the expression level of wild-type cells grown in minimal medium (relative value = 1). Oligonucleotides used for qRT-PCR analyses are listed in S3 Table.

### Computational methods

#### Homology Modeling of S. pombe Gad8

The sequence of *S*. *pombe* Gad8 (accession number: Q9P7J8) was downloaded from the UniprotKB/Swiss-Prot server [68]. Available three-dimensional structures of close homologues in the Protein Data Bank (PDB) were identified using BLAST [69]. The crystal structure of inactive (un-phosphorylated) SGK1 in complex with AMP-PNP with a resolution of 1.9Å (PDB code: 2R5T) [70] was found as the most suitable template for homology modeling. This template lacks the αC-helix region and the C-terminal extension regions, which were modeled based on the crystal structures of AKT1 kinase (PDB code: 6NPZ) [71] and PKCα (PDB code: 4RA4). Multiple sequence alignment between the target and templates were derived using CLUSTALW [72]. After visual inspection of the alignment, models were generated using the default parameters in MODELLER v. 9.23 [73]. The model was built in the presence of AMP-PNP and a Mg2^+^ ion. The models were ranked using DOPE energy score, which was calculated by the "evaluate model" python script in MODELLER. The highest ranked model was tested for stereochemical quality using Procheck [74] and was selected for subsequent analysis.

#### System preparation

The mutation K263C and the phosphorylation at positions S527 and S546 were introduced into Gad8 using Schrodinger’s Maestro 11.2. All the structures were prepared using the Protein Preparation Wizard as implemented in Maestro 11.2. This protocol adds missing hydrogen atoms considering a pH value of 7.0 ± 1.0, optimizes the hydrogen bond network, and performs restrained minimization. For further calculations, the ATP analogue (AMP-PNP) was replaced by ATP. Next, each construct (Gad8, Gad8-K263C and Gad8-pS527/pS546) were submerged in TIP3P water model in octahedron box with an additional 15-Å extension along each axis of the protein. Sodium and chloride ions were added to the water phase in order to neutralize the system and to obtain a salt concentration of 0.15 M.

#### Molecular dynamics simulations

All MD simulations were performed with the GROMACS 5.0.6 package [75] using the Amber14 force field [76]. Force field parameters for ATP were obtained by using the ANTECHAMBER module in AMBER [77]. The partial atomic charges for ATP were derived using quantum-mechanical (QM) single point energy calculation. This calculation was performed with Jaguar [78], as implemented in Schrodinger’s Maestro 11.2, using the 6-31G* basis set and the HF level of theory. The simulations were conducted in periodic boundary conditions (PBC) with particle-mesh Ewald (PME) [79] electrostatics with 8 Å cutoff for long range electrostatics. First, the simulated systems were energy-minimized with the steepest descent minimization algorithm in order to remove van der Waals clashes within the systems. Following the minimization, each one of the systems was equilibrated at two stages: (1) 2 ns of simulation in NVT ensemble (constant temperature); (2) 2 ns of simulation in NPT ensemble (constant pressure). During the equilibration stages the heavy atoms of the protein and ATP were restrained. Finally, a 50 ns the production phase was carried out for each system under constant temperature and pressure of 310K and 1 bar, respectively. A time step of 2 fs was applied. Each of the systems were simulated three times, each time starting from a different random seed.

#### Data analysis

The resulting trajectories were visually inspected using VMD 1.9.3 software [80]. The stability of the resulting trajectories and the average mobility of all residues were tested based on the root mean square deviation (RMSD) of the backbone atoms of the protein from the initial structure and on the root mean square fluctuations (RMSF), respectively. RMSD and RMSF were calculated using the RMS and RMSF utilities of the GROMACS package, respectively.

## Supporting information

S1 Fig Isolation Tor1-independent alleles of *gad8*. ***A*.** *gad8-m1*/*m2/m3*, suppressed the temperature or osmotic stress sensitivities of *tor1*-*L2045D* or Δ*tor1* cells. Stress sensitivities were evaluated by growth on solid EMM media. Empty pREP81 vector or pREP81-*gad8*^+^ plasmids were used as negative control. pREP1-*gad8*^+^ was used as positive control. ***B*.** Schematic representation of the ORF of *gad8* mutant alleles that were isolated in a screen for Tor1-independent alleles.
(TIF)

S2 Fig The *tor1*-independent *gad8* mutations suppress defects associated with loss of function of TORC2. ***A-B***, Stress sensitivities were evaluated by serial dilutions of the indicated transformant strains on solid EMM media. ***C*,** Mating efficiencies of wild type (WT), Δ*tor1* or Δ*ste20* strains transformed with the indicated plasmids. The results are the mean values of three independent experiments.
(TIF)

S3 Fig Gad8-K263C is phosphorylated at S546 in the absence of Tor1. ***A-B***, Western blot analysis of protein extracts isolated from strains expressing no-tag Gad8, Gad8-HA or Gad8-K263C-HA. Cells were grown to mid-log phase and left untreated in YES medium, or shifted for 1 h to EMM containing no carbon source (-Glu), YES containing 1M KCl or 1 M NaCl (A), or shifted for 1 h to YES containing 12 mM hydroxyurea (HU) or 40 μM camptothecin (CPT) (B). Phosphorylation of Gad8 at S456 was detected with anti-Gad8-S546-P phsophospecific antibodies (S546-P). Anti-actin antibody was used as loading control.
(TIF)

S4 Fig The Q298 residue in Gad8 is conserved throughout evolution. Alignment of the amino acid sequences surrounding the point mutations in several members of the AGC kinases in *S*. *pombe*, *S*. *cerevisiae* and human. The *tor1*-independent *gad8* mutations are in red.
(TIF)

S5 Fig Sensitivity to genotoxic stress depends on the kinase activity of Gad8 and its phosphorylation by TORC2. ***A*.** Cells carrying a *gad8* kinase-dead allele, *gad8*-*K259R* are sensitive to DNA damaging conditions. Stress sensitivities were evaluated by growth on solid YES media. Plates were incubated for 3 days. ***B*.** Cells carrying *gad8* mutant alleles that cannot be phosphorylated by TORC2 are sensitive to genotoxic stress. Stress sensitivities were evaluated by serial dilutions as above.
(TIF)

S6 Fig *gad8-K263C*, *gad8-K263A*, *gad8-K263*R or *gad8-T260*, but not *gad8-S527D/S546D*, confer sensitivity to DNA damaging conditions. ***A*,** Plasmids containing *gad8-K263C* or *gad8-T260* are unable to suppress genotoxic sensitivity in TORC2 mutant cells. Stress sensitivities were evaluated by serial dilutions of cells onto EMM media ***B*,** *gad8-K263A* or *gad8-K263R* confer genotoxic stress sensitivity in wild type cells, while suppressing high temperature, osmotic stress or low-glucose sensitivities in Δ*tor1* cells. in wild type cells. Stress sensitivities were evaluated by serial dilutions of cells onto EMM media. ***C*,** Mutant alleles of *gad8* that mimic constitutive phosphorylation at S527 and S546 suppress high temperature, osmotic stress, low glucose stress, as well as genotoxic stress. pR3C-*gad8-S546D* and pR3C-*gad8-S527D/ S546D* are a kind gift from A. Yamashita, National Institute for Basic Biology, Okazaki, Japan.
(TIF)

S1 Table Strains used in this study. (DOCX)

S2 Table Oligonucleotides used for introducing point mutations in *gad8*^+^. (DOCX)

S3 Table Oligonucleotides used for qRT-PCR analyses. (DOCX)
