# Supplementary figures and images for: TOR Complex 2- independent mutations in the regulatory PIF pocket of Gad8AKT1/SGK1 define separate branches of the stress response mechanisms in fission yeast
Source: PLoS Genet. 2020 Nov 2;16(11):e1009196. doi: 10.1371/journal.pgen.1009196 (PMC7660925; doi:10.1371/journal.pgen.1009196)

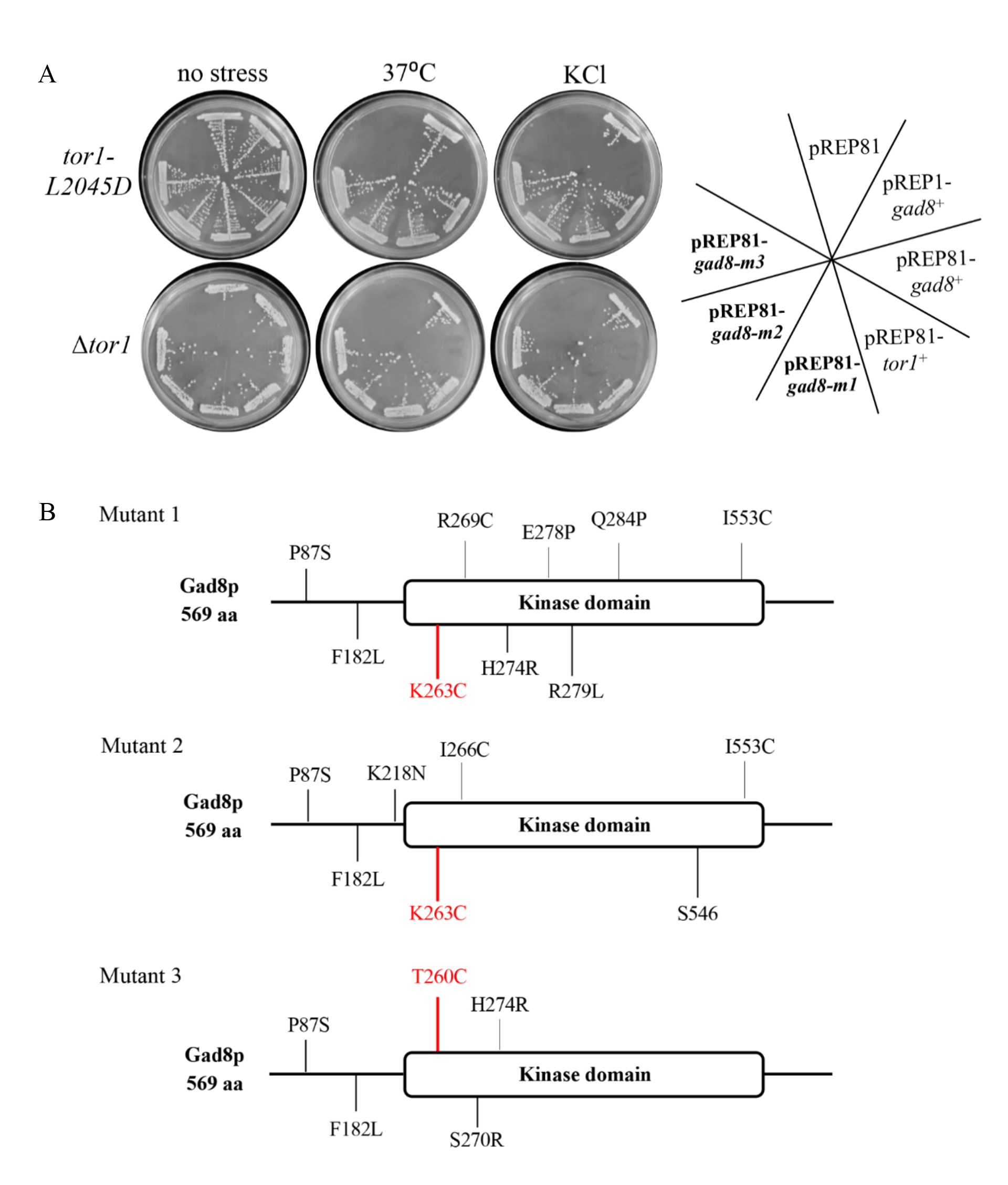

Supplement: S1 Fig — A. gad8-m1/m2/m3, suppressed the temperature or osmotic stress sensitivities of tor1-L2045D or Δtor1 cells. Stress sensitivities were evaluated by growth on solid EMM media. Empty pREP81 vector or pREP81-gad8+ plasmids were used as negative control. pREP1-gad8+ was used as positive control. B. Schematic representation of the ORF of gad8 mutant alleles that were isolated in a screen for Tor1-independent alleles. (TIF) [file pgen.1009196.s001.tif]

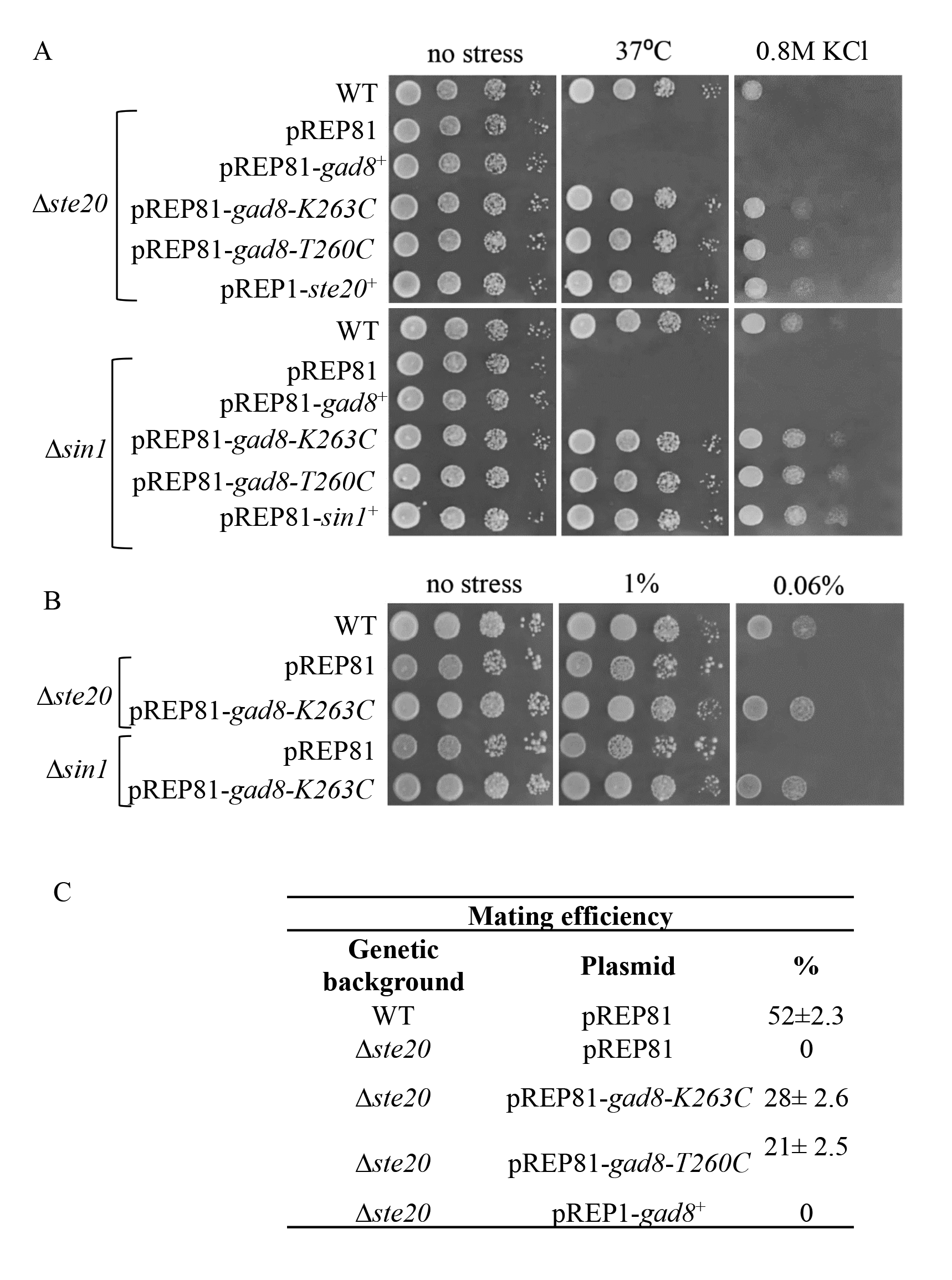

Supplement: S2 Fig — A-B, Stress sensitivities were evaluated by serial dilutions of the indicated transformant strains on solid EMM media. C, Mating efficiencies of wild type (WT), Δtor1 or Δste20 strains transformed with the indicated plasmids. The results are the mean values of three independent experiments. (TIF) [file pgen.1009196.s002.tif]

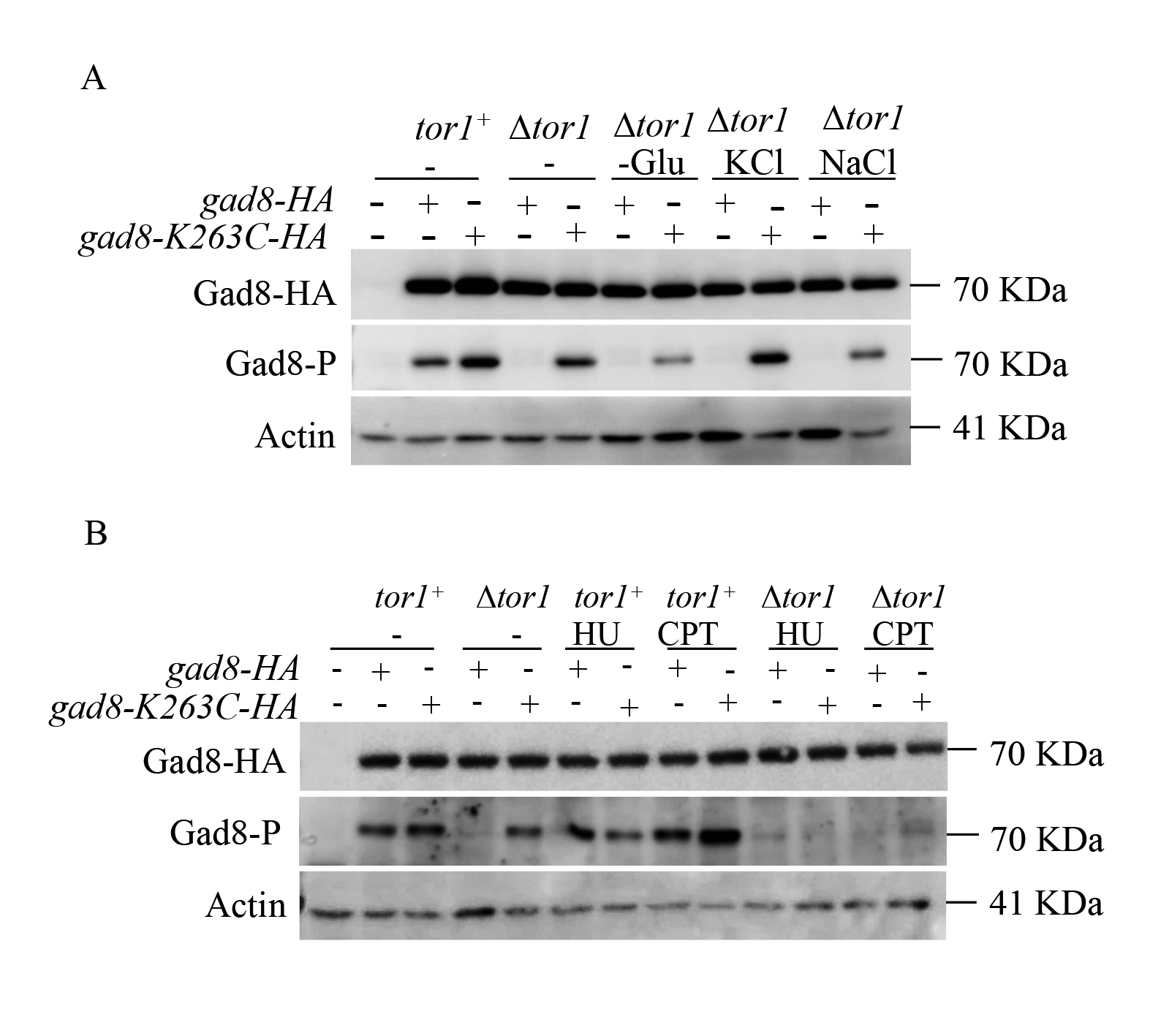

Supplement: S3 Fig — A-B, Western blot analysis of protein extracts isolated from strains expressing no-tag Gad8, Gad8-HA or Gad8-K263C-HA. Cells were grown to mid-log phase and left untreated in YES medium, or shifted for 1 h to EMM containing no carbon source (-Glu), YES containing 1M KCl or 1 M NaCl (A), or shifted for 1 h to YES containing 12 mM hydroxyurea (HU) or 40 μM camptothecin (CPT) (B). Phosphorylation of Gad8 at S456 was detected with anti-Gad8-S546-P phsophospecific antibodies (S546-P). Anti-actin antibody was used as loading control. (TIF) [file pgen.1009196.s003.tif]

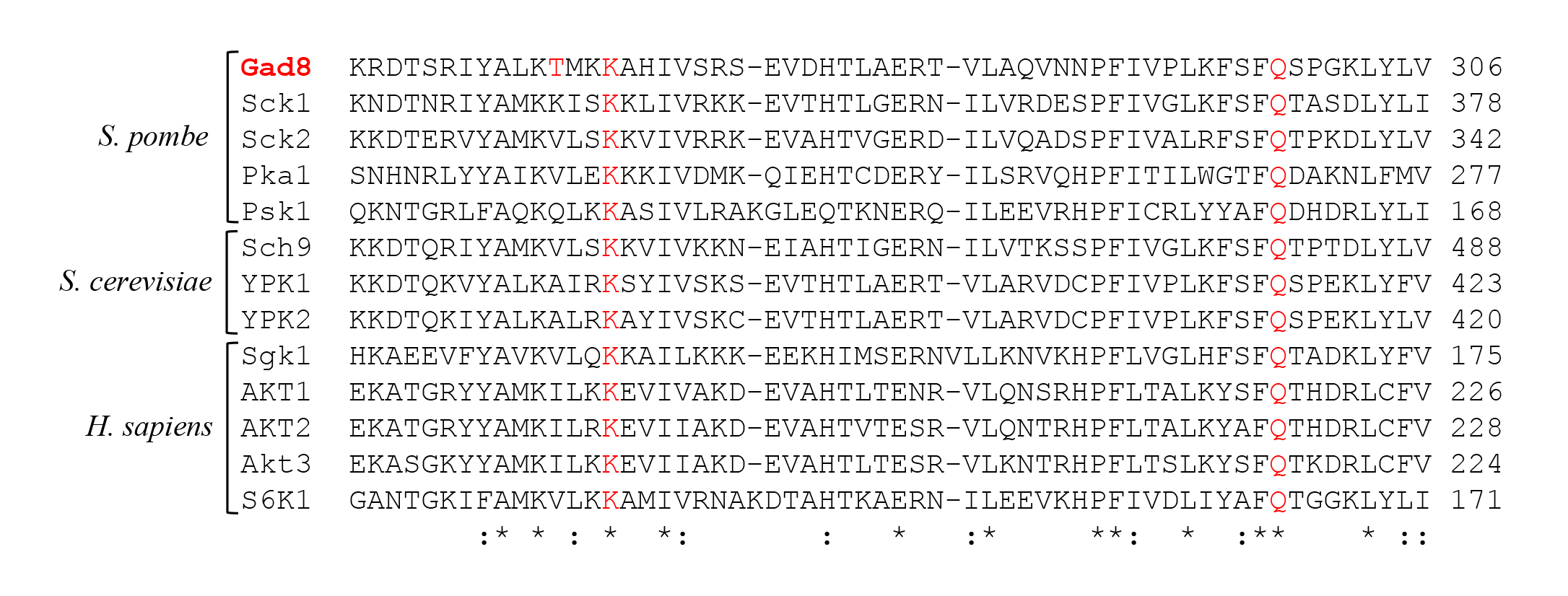

Supplement: S4 Fig — Alignment of the amino acid sequences surrounding the point mutations in several members of the AGC kinases in S. pombe, S. cerevisiae and human. The tor1-independent gad8 mutations are in red. (TIF) [file pgen.1009196.s004.tif]

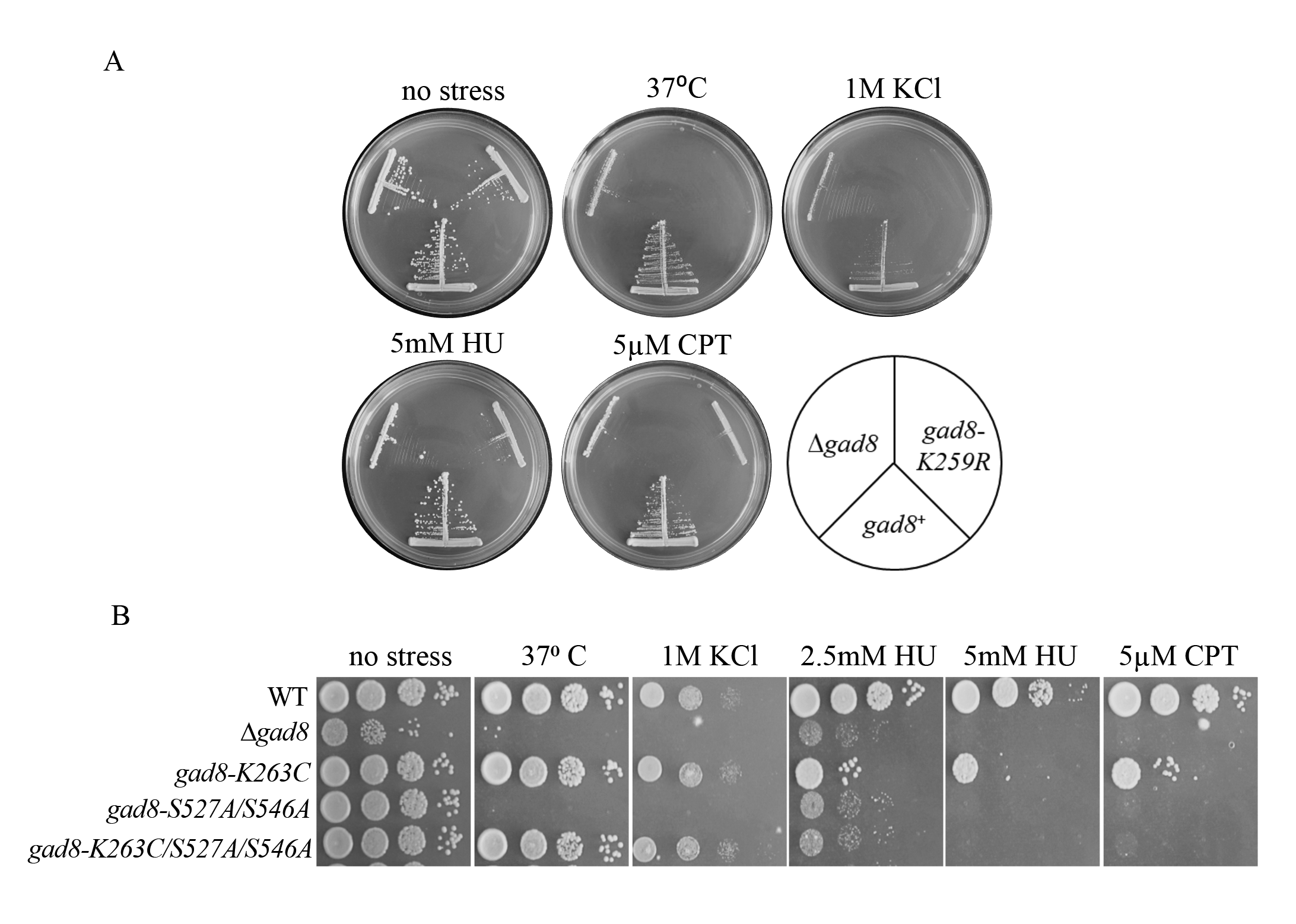

Supplement: S5 Fig — A. Cells carrying a gad8 kinase-dead allele, gad8-K259R are sensitive to DNA damaging conditions. Stress sensitivities were evaluated by growth on solid YES media. Plates were incubated for 3 days. B. Cells carrying gad8 mutant alleles that cannot be phosphorylated by TORC2 are sensitive to genotoxic stress. Stress sensitivities were evaluated by serial dilutions as above. (TIF) [file pgen.1009196.s005.tif]

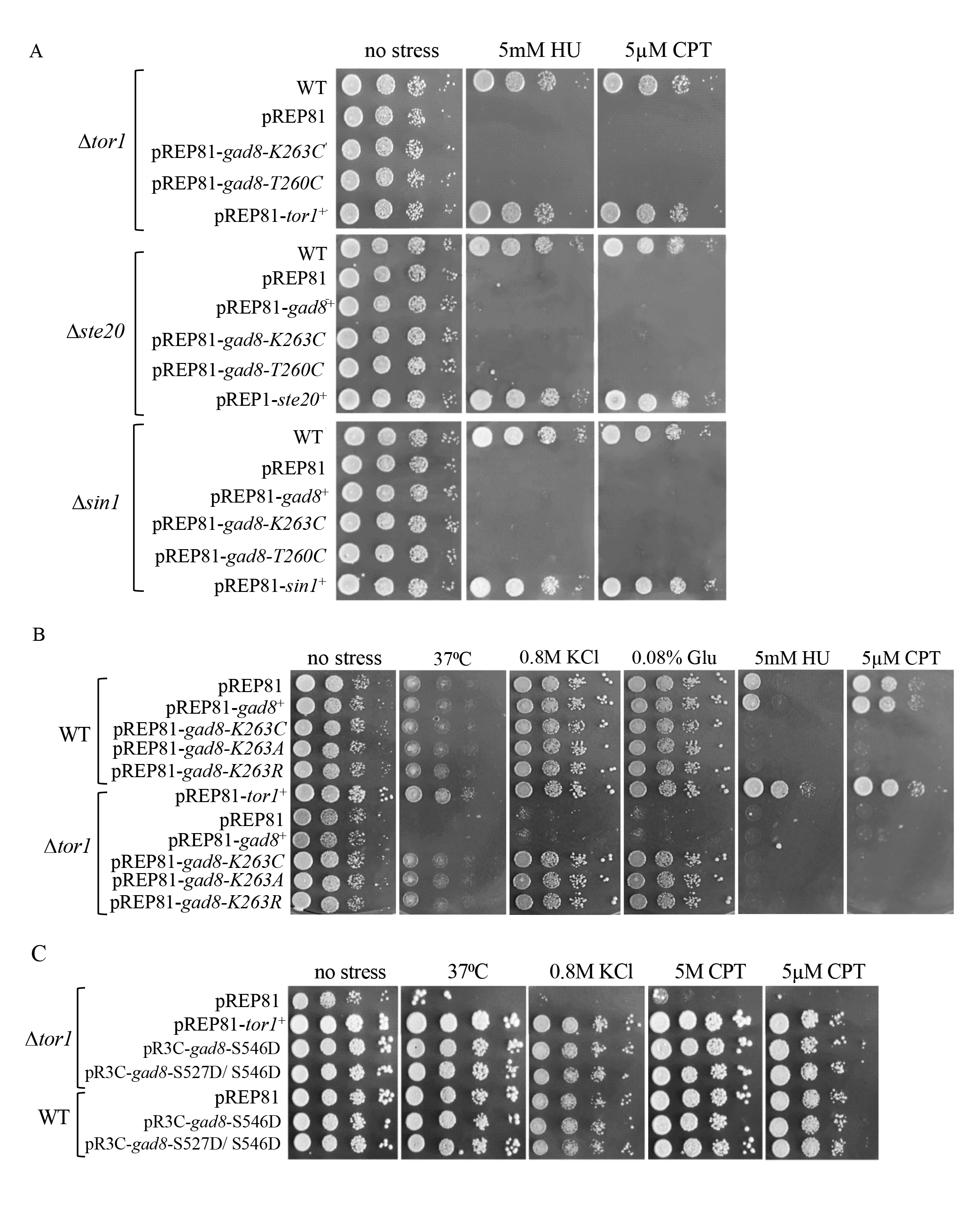

Supplement: S6 Fig — A, Plasmids containing gad8-K263C or gad8-T260 are unable to suppress genotoxic sensitivity in TORC2 mutant cells. Stress sensitivities were evaluated by serial dilutions of cells onto EMM media B, gad8-K263A or gad8-K263R confer genotoxic stress sensitivity in wild type cells, while suppressing high temperature, osmotic stress or low-glucose sensitivities in Δtor1 cells. in wild type cells. Stress sensitivities were evaluated by serial dilutions of cells onto EMM media. C, Mutant alleles of gad8 that mimic constitutive phosphorylation at S527 and S546 suppress high temperature, osmotic stress, low glucose stress, as well as genotoxic stress. pR3C-gad8-S546D and pR3C-gad8-S527D/ S546D are a kind gift from A. Yamashita, National Institute for Basic Biology, Okazaki, Japan. (TIF) [file pgen.1009196.s006.tif]
