# Supplementary material for: TOR Complex 2- independent mutations in the regulatory PIF pocket of Gad8AKT1/SGK1 define separate branches of the stress response mechanisms in fission yeast
Source: PLoS Genet. 2020 Nov 2;16(11):e1009196. doi: 10.1371/journal.pgen.1009196 (PMC7660925; doi:10.1371/journal.pgen.1009196)
Supplement: S1 Table — (DOCX) [file pgen.1009196.s007.docx]

S1 Table. Strains used in this study.

| **Strain** | **Genotype** | **Source** |
| --- | --- | --- |
| TA2 | *leu1-32 ura4-D18 ade6-M210 h^-^* | Laboratory stock |
| TA16 | *leu1-32 ura4-D18 ade6-M216 h^90^* | Laboratory stock |
| TA120 | *leu1-32 ura4-D18 tor1::ura4^+^ h^+^* | Laboratory stock |
| TA157 | *leu1-32 ura4-D18 ade6-216 tor1::ura4^+^ h^90^* | Laboratory stock |
| TA808 | *gad8::ura ade6-M216 leu1-32 ura4-D18 h^90^* | M. Yamamoto |
| TA813 | *ste20::ura4^+^ ade6-M216 leu1-32 ura4-D18 h^90^* | M. Yamamoto |
| TA820 | *Chk1:9myc2HA6HA:ura4^+^ leu1-32 ura4-D18 h^-^* | P. Russell |
| TA880 | *tor1::his chk1:9myc2HA6HA:ura4^+^ leu1-32 ura4-D18* | Laboratory stock |
| TA913 | *sin1::kanMX6 leu1-32 ura4-D18 h^-^* | K. Shiozaki |
| TA1029 | *gad8::ura4^+^ ade6-M216 leu1-32 ura4-D18 h^-^* | M. Yamamoto |
| TA1124 | *gad8-S572A/S546A-6HA<<kanMX gad8::ura ade6-M210 leu1 ura4 h^90^* | YGRC* |
| TA1125 | *gad8::ura4<<kanr-gad8-6HA tor1::ura4 ade6-M210 leu1. (ura4-D18?) h^90^* | YGRC |
| TA1126 | *gad8::ura4<<kanMX-gad8-6HA ade6-M210 leu1-32 ura4^+^(?) h^90^* | YGRC |
| TA1375 | *gad8::ura4<<kanMX-gad8K259R-6HA ade6-M210 leu1 ura4-D18, h90* | YGRC |
| TA3079 | [*tor1*](http://www.pombase.org/spombe/result/SPBC30D10.10C)*-L2045D:hygR h^-^* | YGRC |
| TA3110 | [*tor1*](http://www.pombase.org/spombe/result/SPBC30D10.10C)*-L2045D:hygR leu1-32 h^+^* | This study |
| TA3738 | *tor1::ura4^+^ ura4-D18 gad8-K263C<< kanMX ade6M210 leu1-32 h^90^* | This study |
| TA3775 | *gad8-K263C leu1-32 ura4-D18 ade6-M210 h^-^* | This study |
| TA4036 | [*gad8*](http://www.pombase.org/spombe/result/SPCC24B10.07)::[*ura4*](http://www.pombase.org/spombe/result/SPCC330.05c)<<*kanr*-[*gad8*](http://www.pombase.org/spombe/result/SPCC24B10.07)*T387A*-*6HA* [*ade6*](http://www.pombase.org/spombe/result/SPCC1322.13)-*M210*[*leu1*](http://www.pombase.org/spombe/result/SPBC1A4.02c) [*ura4*](http://www.pombase.org/spombe/result/SPCC330.05c)*-D18 h*^90^ | YGRC |
| TA4566 | *gad8-S527A/S546A/K263C-6HA<<kanMX gad8::ura ade6-M210 leu1 ura4 h^90^* | This study |
| TA4595 | *gad8-K263C<<kanMX-gad8-6HA ade6-M210 leu1-32 ura4^+^ h^90^* | This study |
| TA4596 | [*gad8*](http://www.pombase.org/spombe/result/SPCC24B10.07)-*K263C*<<*kanr*-[*gad8*](http://www.pombase.org/spombe/result/SPCC24B10.07)*T387A*-*6HA* [*ade6*](http://www.pombase.org/spombe/result/SPCC1322.13)-*M210*[*leu1*](http://www.pombase.org/spombe/result/SPBC1A4.02c) [*ura4*](http://www.pombase.org/spombe/result/SPCC330.05c)*-D18 h*^90^ | This study |
| TA4598 | *gad8-K263C Chk1:9myc2HA6HA:ura4^+^ leu1-32 ura4-D18 h^-^* | This study |
| TA4599 | *leu1-32 ura4-D18 ade6-M210/ade6M216* h^-^ | This study |
| TA4601 | *gad8::ura4/gad8::ura4 ade6-M216/ade-M210 leu1 ura4-D18* h^-^ | This study |
| TA4602 | *gad8-K263C/gad8-K263C leu1-32 ade6-M216/ade6-M210 h^-^* | This study |
| TA4603 | *gad8-K263C/gad8^+^ leu1-32 ade6-M216/ade6-M210 h^-^* | This study |
| TA4620 | *gad8-K263C ade6-M216 leu1-32 ura4-D18 h^90^* | This study |

*YGRC- Yeast Genetic Resource Center, Japan
