# Supplementary material for: TOR Complex 2- independent mutations in the regulatory PIF pocket of Gad8AKT1/SGK1 define separate branches of the stress response mechanisms in fission yeast
Source: PLoS Genet. 2020 Nov 2;16(11):e1009196. doi: 10.1371/journal.pgen.1009196 (PMC7660925; doi:10.1371/journal.pgen.1009196)
Supplement: S2 Table — (DOCX) [file pgen.1009196.s008.docx]

**S2 Table.** Oligonucleotides used for introducing point mutations in *gad8*^+^.

| **Name** | | **Sequence** |
| --- | --- | --- |
| *gad8*-*K263A* | #1797 F | CTTTAAAAACTATGAAAGCCGCCCACATTGTATCTCGCAGTGAAG |
|  | #1796 R | CATAGATACGAGAAGTGTCACGC |
| *gad8*-*K263R* | #1801 F | CTTTAAAAACTATGAAAAGGGCCCACATTGTATCTCGCAGTGAAG |
|  | #1796 R | CATAGATACGAGAAGTGTCACGC |
| *gad8*-*Q298L* | #1820 F | AGCTGTATCTTGTCCTGG |
|  | #1807 R | TACCGGGAGAGAGAAAGGAGAACT |
