# Supplementary material for: TOR Complex 2- independent mutations in the regulatory PIF pocket of Gad8AKT1/SGK1 define separate branches of the stress response mechanisms in fission yeast
Source: PLoS Genet. 2020 Nov 2;16(11):e1009196. doi: 10.1371/journal.pgen.1009196 (PMC7660925; doi:10.1371/journal.pgen.1009196)
Supplement: S3 Table — (DOCX) [file pgen.1009196.s009.docx]

**S3 Table.** Oligonucleotides used for qRT-PCR analyses.

| **Name** | **Target** | **Sequence** |
| --- | --- | --- |
| #1210 *cdt2*^+^ F | *cdt2* | TGAGACTGGAGCTCTTGAGCTGTT |
| #1211 *cdt2^+^* R | *cdt2* | TAGCATTGTTGTGAGCAAGCCAGC |
| #1212 *cdc22*^+^ F | *cdc22* | TGCAACGTGTTGAACGTAACGAGC |
| #1213 *cdc22*^+^ R | *cdc22* | AGGTAATGAACGACGACCACGGTT |
| #1214 *cdc18*^+^ F | *cdc18* | GTAGGCATGCAATTGAACTTGCGG |
| #1215 *cdc18*^+^ R | *cdc18* | TCATAGCAGATGTCGCTCGGACAA |
